# Coupled cluster theory on modern heterogeneous supercomputers

**DOI:** 10.3389/fchem.2023.1154526

**Published:** 2023-06-14

**Authors:** Hector H. Corzo, Andreas Erbs Hillers-Bendtsen, Ashleigh Barnes, Abdulrahman Y. Zamani, Filip Pawłowski, Jeppe Olsen, Poul Jørgensen, Kurt V. Mikkelsen, Dmytro Bykov

**Affiliations:** ^1^ Oak Ridge National Laboratory, Oak Ridge, TN, United States; ^2^ Department of Chemistry, University of Copenhagen, Copenhagen, Denmark; ^3^ Department of Chemistry and Biochemistry and Center for Chemical Computation and Theory, University of California, Merced, CA, United States; ^4^ Department of Chemistry and Biochemistry, Auburn University, Auburn, AL, United States; ^5^ Department of Chemistry, Aarhus University, Aarhus, Denmark

**Keywords:** coupled cluster theory, divide-expand-consolidate coupled cluster framework, cluster perturbation theory, excitation energies, tetrahydrocannabinol, deoxyribonucleic acid

## Abstract

This study examines the computational challenges in elucidating intricate chemical systems, particularly through *ab-initio* methodologies. This work highlights the Divide-Expand-Consolidate (DEC) approach for coupled cluster (CC) theory—a linear-scaling, massively parallel framework—as a viable solution. Detailed scrutiny of the DEC framework reveals its extensive applicability for large chemical systems, yet it also acknowledges inherent limitations. To mitigate these constraints, the cluster perturbation theory is presented as an effective remedy. Attention is then directed towards the CPS (D-3) model, explicitly derived from a CC singles parent and a doubles auxiliary excitation space, for computing excitation energies. The reviewed new algorithms for the CPS (D-3) method efficiently capitalize on multiple nodes and graphical processing units, expediting heavy tensor contractions. As a result, CPS (D-3) emerges as a scalable, rapid, and precise solution for computing molecular properties in large molecular systems, marking it an efficient contender to conventional CC models.

## 1 Introduction

Over the past 6 decades, the field of computational chemistry and molecular modeling has aimed to solve for the energy and expectation values of wave functions for atomic and molecular systems. In the exact limit, the non–relativistic electronic contribution to the total energy of a many-body system can be obtained by finding the exact solution to the N-electron Schrödinger equation. However, due to the challenges in solving atomic and molecular systems composed of more than few electrons in orbitals with angular momentum *l* ≥ 1, many numerical approximations have been rather crude. To reduce the mathematical complexity associated with solving multi-electron molecular systems, which often requires modeling a 3*N* dimensional space, many computational chemistry approximations have opted for partial or total neglect of electron-electron correlation (Tew et al., 2007) and relativistic effects (Pyykkö, 2012; Liu, 2020). Furthermore, in many cases, the neglect of inner-core electrons, the acceptance of insufficient Born-Oppenheimer approximation, and the disregard of chemically unique tailored basis sets in favor of a one-size-fits-all approach have become routine in many quantum chemistry calculations (Woolley and Sutcliffe, 1977; Combes et al., 1981; Davidson and Feller, 1986; Cederbaum, 2008; Nagy and Jensen, 2017). In general, the larger the system with respect to the number of electrons, the cruder the approximations become. It can be argued that, in a way, the current state of many computational chemistry methodologies is as paradoxical as it was 60 years ago. For small systems, where very accurate experiments are often readily accessible, we find increasingly powerful and reliable quantum chemical computational methods and techniques being developed. However, for complex chemical systems like those typically found in biological applications where accurate experimental data requires unambiguous interpretation and quantitative predictions from theoretical models, one often finds an extensive application of low-accuracy quantum chemical methods. Only a few decades ago, modeling of biochemical systems was limited to empirical and semiempirical methods, where approximations such as the Hückel model and methods based on the neglect of diatomic differential overlap were either presented or used as reliable, despite strong evidence to the contrary obtained from studies on small systems (Elstner et al., 2000; Hagebaum-Reignier et al., 2007; Kutzelnigg, 2007; Yates, 2012; Elstner and Seifert, 2014; Thiel, 2014; Christensen et al., 2016; Dral et al., 2019).

Recently, the development of many branches of sciences has been accelerated by the use of machine learning (ML) models that contain a large number of parameters, which are weighted and tuned during the training process. As a result, ML models have had a transformative impact on the chemical sciences. For molecular applications, the design and assembly of a compelling ML model often requires a significant investment of computational resources not only for algorithm processing but also for generating accurately labeled data and ground truths for analysis and pattern inferences necessary for the training. Indeed, once attained, ML models can drastically reduce the computational time for routine tasks in molecular modeling, thereby amplifying the amount of data that can be generated for a given dataset. However, it is important to note that ML models usually fail to generate the insights necessary for explaining the electronic structure of molecules. Many of the reported works on molecular applications of ML models propose that these models are not just proxies but computational ends for theoretical molecular quantum chemistry methods. Nonetheless, many of these methods rely on correlations between families of molecules, where basic parameters such as the molecular topology, molecular local descriptors, and narrow electronic property classification of relationships are emphasized (Haghighatlari and Hachmann, 2019; Corzo et al., 2021; Fung et al., 2021; Keith et al., 2021; Unke et al., 2021). As a consequence, the use of current ML models as an alternative for *ab-initio* quantum-chemical techniques may be less relevant when elucidating molecular phenomena for which unprecedented electron correlation effects and possibly the interactions between electronic and nuclear degrees of freedom play a fundamental role, e.g., redox processes, chemical reactions, photochemical processes, organometallic catalysis, *etc.*

Computational practitioners working in molecular modeling should be mindful that a given model is only useful within a given range of applications; outside that range, although the theory or data sets behind the computational model may be correct, the model might not be necessarily useful. Note that there is a tendency to use techniques that were developed for small chemical systems to study large systems, rather than to search for new methods devised specifically to deal with large systems. It follows that modeling biological molecules should rely on direct calculations or ML models trained from accurate *ab-initio* quantum chemical approximations. An understanding of the proper applicability range of ML and computational quantum chemistry models is important, since the traditional neglect of quantities such as time, temperature, entropy, spin angular momentum, and correlation, namely, those physical quantities that have been considered basic since the last century to describe complex chemical systems, can lead to erroneous predictions. One-size-fits-all approaches may present an additional complication, as the only tool available may not be suitable for every problem. With this in mind, the cognitive bias of Maslow’s law of the hammer (Maslow, 1966) should be avoided, as reluctance and clemency of this may stifle the advancement of fields such as quantum chemistry, where great efforts to develop new methods and theories are still needed to achieve the deductive and inductive interpretability goals required by experimental molecular sciences.

## 2 Challenges in large systems

The definition of a large molecular system can be approached from two angles: the number of electrons and the size of the molecule, defined by the ensemble of atoms. For instance, Br_2_ contains 70 electrons but only two atoms, making it a small system despite its electron count, while H_10_, H_50_, and H_64_ (Lin et al., 2011; Li et al., 2021; Mitxelena and Piris, 2022) are larger in size yet smaller in electron count than Br_2_. In this review, we adopt a definition of large molecules as a compromise between electron count and molecular size. We focus on medium-to large-sized molecules containing dozens of electrons, with potential applications in biological, biochemical, catalytic, photochemical, and technological materials.

To describe large molecular systems accurately and advance scientific and technological endeavors of quantum chemistry, one must develop and implement computational methodologies always guided by the following question: what is the largest number of atoms for which it is still reasonable and, at times, necessary to request electronic energy contributions from solutions of the Schrödinger equation? Although this question may seem simple and perhaps trivial at first, its answer requires not only an understanding of quantum chemistry models, electron-electron correlation, and scalability but also an understanding of the implications associated with the intrinsic phenomenological nature of chemical processes, error propagation, and the ideas related to chemical accuracy first introduced by S.F. Boys (Boys and Rajagopal, 1966) and later popularized by J.A. Pople (Pople, 1999).

In standard *ab-initio* quantum chemistry simulations, the computational bottleneck primarily stems from the number of basis functions used to represent electrons in the system under study, rather than the number of electrons themselves. Accurate calculations are often obtained by employing a combination of computational approximations designed to yield the exact solution to the electronic Schrödinger equation. In this respect, the exact numerical solution for stationary states of a given Schrödinger equation can be obtained through the configuration interaction (CI) matrix-eigenvalue equation. This matrix-eigenvalue equation and its Hamiltonian representation, expressed in terms of Slater determinants and a sufficiently large orbital basis (where *l* → *∞*), defines what is commonly known as the Full CI (FCI) method, which is utilized to determine FCI energies. In practice, however, CI is an essentially intractable problem, due to its computational demands. Solutions for CI up to a *n* number of excitations (CI-nx) and Full CI (FCI) for *n* ≤ 4 electrons are typically challenging to obtain. Therefore, in many cases, compact basis sets and computationally feasible approximations to the CI solution, such as the Coupled Cluster approach (CC), are often preferred. In general, molecular systems at their equilibrium geometry are known to exhibit rapid convergence to the Full CI solution through the use of the CC hierarchy of approximations.

Quantum chemistry, in pursuit of high accuracy, employs (CC) family of methods to construct multi-electron wavefunctions using the exponential cluster operator and a molecular orbital basis. The CC method is particularly appealing not only for its remarkable accuracy and rapid convergence, but also for its size extensivity and size consistency properties (Bartlett, 2012). In contrast to other computational methods, CC energy remains unitarily invariant with respect to the rotation within occupied and virtual spin-orbital space, respectively. Hence, it is the ideal computational method for accounting for electron correlation and making accurate determinations for medium-sized molecules. With efficient implementation and approximations correlated *ab*-initio quantum chemistry methods like CC can be performed for an upper limit of 200–500 atoms with paired electrons within orbitals with angular momentum *l* ≤ 2. However, despite advances in computational hardware, routine calculations may still be limited to 30–50 atoms. Therefore, large and complex molecular systems, especially those found in biological or biochemical processes, continue to present challenges in computational chemistry. Accurately estimating the molecular electronic energy in these systems requires consideration of both the number of electrons in the atoms and the size of the system, as well as the relationships between internuclear distances and electronic density, decay of overlap integrals, the long-range nature of Coulomb forces, and the conformational flexibility of molecules, among other factors.

Thus, it is of practical importance to develop reliable computational methods that can be used for molecules approaching the system size limit, which is yet to be systematically defined. Possible approaches may include: (1) Employ reasonable approximations to matrix elements at the self-consistent-field (SCF) level. These approximations often require a distinction between the Roothaan Hartree-Fock method and the canonical nature of the SCF method (Löwdin, 1955a; Löwdin, 1955b; Nesbet, 1955). (2) Generate new techniques for integral evaluation and their applicability to new approximations at the SCF and post-SCF level. (3) Formulate post-SCF approximations for obtaining a better description of electron-electron correlation energy. (4) The development of algorithms and techniques that can leverage the inherent parallelism in computational tasks to achieve optimal performance on modern high-performance computing (HPC) architectures.

The field of quantum chemistry and general electronic structure theory has witnessed productive research efforts in these directions, with researchers employing clever computational simplifications within new theoretical frameworks and developing new approaches that can effectively scale computations to larger problem sizes, improving the accuracy and efficiency of simulations. These developments have been well-documented in the literature (Häser, 1993; Ishikawa and Kuwata, 2012; Monari et al., 2013; Helmich and Hättig, 2014; Díaz-Tinoco et al., 2016; Gyevi-Nagy et al., 2019; Mester et al., 2019; Nagy and Kállay, 2019; Ballesteros et al., 2021; Datta and Gordon, 2021; Gyevi-Nagy et al., 2021; Szabó et al., 2021; Abyar and Novak, 2022; Paudics et al., 2022; Semidalas and Martin, 2022).

## 3 Scaling and parallelization of quantum chemistry computations

As both molecular system sizes and computer resources grow larger, efficient computation scaling becomes critical. On commodity computer hardware, which is the basis for the majority of available clusters and HPC resources, an effective strategy would be combining computing power of multiple units, allowing for a lower time-to-solution. This implies effective management of computational tasks in hand. Quantum chemistry calculations often involve tasks with variety of computational costs and dependencies. For example, in the SCF procedure, constructing and diagonalizing the Fock matrix depends on computing and summing up many integrals over basis functions that can vary in size and complexity. Finding the way to expose the parallelism in the SCF procedure and other quantum chemistry methods can yield improved performance on many different kinds of computers, especially modern HPC architectures. However, achieving optimal speedup is challenging, and only very few parallel implementations of quantum chemistry methods can demonstrate it. Domain decomposition and speculative parallelization are general techniques that have proven useful in identifying and designing parallel algorithms for large and complex quantum chemistry calculation tasks (Werner, 1995; González-Escribano et al., 2006; Su et al., 2007; Lipparini et al., 2013; Qiu et al., 2017; Nottoli et al., 2019; Sho and Odanaka, 2019; Jha et al., 2022; Shang et al., 2022; Fedorov and Pham, 2023).

### 3.1 Parallelization strategies

The main parallelization strategies in quantum chemistry computations can generally be categorized into two approaches: fine-grained and coarse-grained parallelism. Fine-grained parallelism focuses on executing numerous small, independent tasks simultaneously across multiple processing elements. This approach is well-suited for tasks with a high degree of data locality, such as dense matrix operations. The matrix and tensor operations are prevalent in quantum chemistry and thus are most often the first target of performance optimization.

Conversely, coarse-grained parallelism divides the computation into larger, independent tasks that can be executed on separate processing elements. Unlike fine-grained parallelism, this approach is more appropriate for situations with fewer, larger tasks that can be executed simultaneously, and that require infrequent communication between them to deliver results. In addition, coarse-grained parallelism is quintessential for tasks that use data that are far apart (i.e., tasks with less data locality). The coarse-grained parallelizm is most often achieved through the reformulation of the underlying theory of a particular quantum chemistry method to expose independent work packets. Examples could be as simple as numerical Hessian evaluation where each nuclear displacement represent independent work packet and all the way to very elaborate CC theory reformulations exposing data locality through physical nature of the quantities to be evaluated.

Many scientific applications achieve both fine-grained and coarse-grained parallelism through shared-memory and distributed-memory parallel programming models. Shared-memory parallelism allows multiple threads of execution to access the same memory space, making it suitable for tasks with a high degree of data locality. In contrast, distributed-memory parallelism uses message-passing techniques for communication between multiple processing elements, each with its own private memory space. Distributed memory is better suited for coarse-grained parallelism, with tightly coupled tasks that communicate frequently. These parallel memory programming models are often supported in quantum chemistry codes by two application programming interfaces: the Message Passing Interface (MPI) and the Open Multi-Processing (OpenMP) application programming interface.

MPI is a standardized, portable message-passing model designed for parallel computing architectures. It is a widely-used distributed-memory parallel programming model that efficiently parallelizes tasks with less data locality, such as the distribution of integrals and the communication of partial results between processing elements. While MPI supports both point-to-point and collective communication, it is generally better suited for coarse-grained parallelism. On the other hand, OpenMP offers the capability to incrementally parallelize a serial program, unlike message-passing models like MPI which typically require an all-or-nothing approach. OpenMP can implement both coarse-grain and fine-grain parallelism. However, many chemistry codes find a hybrid OpenMP and MPI approach most appropriate as it allows for the clear treatment of the two separate levels of parallelism that are often found, coarse-grained and fine-grained, nested within each coarse subdomain.

Quantum chemistry codes often benefit from fine-grained parallelism for tasks such as complete SCF calculations, dense matrix multiplications, DMRG calculations, and other fundamental numerical operations. Graphics Processing Units (GPUs) and OpenMP are the commonly used hardware and programming model, respectively, for implementing this type of parallelism. Coarse-grained parallelism, on the other hand, may be better for partitioning entire component grids onto separate processors. Thus, calculations based on theories such as Density Functional Theory can benefit from this type of parallelization. Recently, fine-grained parallelism has been used to accelerate simulations of quantum circuits on Field Programmable Gate Arrays (FPGAs) (Moawad et al., 2022).

### 3.2 Load balancing

Another crucial aspect for achieving efficient parallelism in large scale supercomputers is load balancing. Load balancing aims to distribute the computational workload evenly among multiple processors or nodes to reduce idle time and communication overhead. This distribution of the workload enhances the performance, efficiency, and scalability of parallel quantum chemistry applications (Nikodem et al., 2014; Ma et al., 2023). Thus, the proper use of the load balancing technique is essential for many production codes and calculations. Load balancing becomes specially important when coarse-grained parallelism is employed. Static Load Balancing (SLB) and Dynamic Load Balancing (DLB) are two types of load balancing techniques that vary depending on whether the workload distribution is fixed or adaptive during the execution of the computation (Ali and Khan, 2012; Patil and Shedge, 2013; Nikodem et al., 2014).

The SLB approach divides the computational work evenly among processing elements before the execution. Thus, in many quantum chemistry codes, SLB is often used to distribute the number of basis functions or integrals equally across processing units. However, although SLB may be effective for many parts of a production code, it may not always lead to optimal load balancing as workloads may vary across tasks. On the other hand, the DLB approach continuously monitors the computational workload of each processing element during execution and redistributes it to balance the load across all processing elements.

On the other hand, the DLB approach involves continuously monitoring the computational workload of each processing element during execution and redistributing tasks as needed to maintain an even distribution of work. For instance, the work-stealing algorithm allows idle processors to steal tasks from busy processors, which can reduce idle time and communication overhead in parallel systems. This algorithm has been shown to be favorable for DFT calculations (Nikodem et al., 2014).

Another algorithm that has been shown to be advantageous in quantum chemistry calculations is the inspector/executor load balancing algorithm. This approach involves two phases: an inspector phase that analyzes the task dependencies and assigns them to the compute units, and an executor phase that performs the tasks in parallel. By reducing the synchronization and contention costs of the parallel system, this algorithm is applicable to any application requiring load balance where reasonable estimations of computational kernel execution times are available. Furthermore, this algorithm reduces the overhead from centralized dynamic load balancing in codes such as NWChem’s Tensor Contraction Engine (TCE) (Ozog et al., 2013).

Load balancing plays a crucial role in efficient parallelization of quantum chemistry computations across multiple computing units, ensuring that no single unit bears too much demand. DLB algorithms are well-suited for fine-grained parallelism in quantum chemistry codes and are designed to adapt to changing workloads by distributing traffic based on real-time conditions. However, they can add communication overhead and slow down the system. SLB algorithms, on the other hand, use fixed rules and are better suited for coarse-grained parallelism in which larger, independent tasks are executed on separate computing units. Therefore, when implementing codes in distributed computing systems for quantum chemistry applications, it is important to carefully consider the trade-offs between DLB and SLB algorithms.

### 3.3 Tensor decompositions in quantum chemistry

The use of parallelization techniques in combination with tensor decomposition methods has proven to be highly effective for computational codes (Boudehane et al., 2022). Tensor decomposition techniques, for example, have proven to be successful in solving various quantum chemistry problems, such as calculating molecular wavefunctions (Altman et al., 2021), density matrices (Hoy and Mazziotti, 2015; Khoromskaia and Khoromskij, 2015), and electronic excitation energies (Xie et al., 2009). In CC theory the electronic wave function of molecules is represented as high-dimensional tensor. Efficient manipulation of the tensors is, thus, of crucial importance. One popular technique commonly used for this purpose is tensor decompositions. Tensor decompositions enable higher-order tensors (multidimensional arrays) to be represented as a combination of lower-order tensors, such as vectors or matrices, which can simplify the complexity and dimensionality of the tensor, uncovering patterns and structures, that can facilitate efficient parallelizations. Third-order tensors, which arise naturally as the outer product of a matrix and a vector during the calculation of spectroscopic properties, offer a practical example. The polarizability tensor is a third-order tensor that describes how a molecule responds to an electric field. Representing the fact that molecules can be polarized to varying extents in different directions, the polarizability tensor is usually represented by a 3 × 3 tensor, denoted as ***α***_*ij*_, where *i*, *j* = *x*, *y*, *z*. Standard manipulations are often challenging to perform on this tensor. Thus, the decomposition of this tensor into vector or matrix components can often reveal critical information about the molecule’s electronic structure, and facilitate efficient parallelization of codes.

One common way to decompose a third-order tensor is to use the CANDECOMP/PARAFAC (CP) decomposition, which expresses the tensor as a sum of rank-one tensors (outer products of three vectors) (Vannieuwenhoven et al., 2015). Although this decomposition has some advantages that may be exploited in quantum chemistry applications such as uniqueness, interpretability, and sparsity, it also presents some challenges such as finding the optimal number of rank-one tensors and solving the nonlinear optimization problem (Stegeman, 2006; Favier and de Almeida, 2014; Battaglino et al., 2018). Canonical polyadic decomposition (CPD) (Hitchcock, 1927; Carroll and Chang, 1970; Qiu et al., 2021), Tucker decomposition (Tucker, 1966), and tensor-train decomposition (TTD) (Oseledets, 2011) are additional tensor decomposition tools that have shown potential for computation on molecular systems. For example, the tensor-train decomposition has been used to compress the wave-function tensors in the quantum chemical calculations of large molecules. By representing the wavefunction in a compressed tensor-train format, it becomes possible to perform calculations for larger systems with reduced computational cost. Similarly, the canonical polyadic decomposition has been employed in the study of molecular properties, such as dipole moments and polarizabilities, by compressing the high-dimensional tensors associated with these properties (Phan et al., 2013).

Nowadays, tensor libraries have been integrated into popular chemistry computational software packages, such as Linear-Scaling Dalton (LSDalton) and North West Computational Chemistry (NWChem), to enhance their performance in CC calculations. NWChem’s TCE (tensor contraction engine) module enables the implementation of TCE-generated code for efficient coupled cluster calculations, including CCSD (coupled cluster singles and doubles) and other methods. LS-Dalton has Scalable Tensor Library, developed to execute tensor contractions in the CC parts of the code. There are also a number of stand alone libraries available to manipulate tensors in quantum chemistry software.

Despite the advancements made using tensor contractions and decompositions in quantum chemistry, there are still challenges to overcome. The choice of decomposition technique, as well as the determination of appropriate rank and truncation parameters, can have a significant impact on the accuracy and efficiency of the calculations. Additionally, the development of robust and efficient algorithms for tensor decompositions is an ongoing area of research (Kolda and Bader, 2009). As these challenges are addressed, tensor decompositions are expected to play an increasingly important role in the development of molecular codes.

### 3.4 Embarrassingly parallel quantum chemistry tasks

In the realm of quantum chemistry, some theoretical methods are considered *embarrassingly parallel* or *trivially parallelizable*. This means that tasks within a computation can be easily divided into independent sub-tasks, that require minimal to no communication or data exchange and coordination between them during execution (Foster, 1995). As a result, these tasks can be seamlessly parallelized, and executed concurrently, without significant alterations to the communication between processing units.

Embarrassingly parallel tasks often arise in the context of evaluating integrals, such as the computation of Hartree-Fock exchange integrals for large basis sets (Titov et al., 2013; Pinski and Neese, 2018), or when solving for quantities like single-point energy calculations, geometry optimizations, and molecular dynamics simulations. In these cases, calculations for each task can be performed independently, with the final result obtained by aggregating or combining individual outcomes. These frameworks allow for parallelization across multiple processing elements, including CPUs or GPUs, enabling larger and more complex problems to be tackled more efficiently.

Unfortunately, the majority of quantum chemistry methods are not easily parallelizable. Certain methods involve complex dependencies between sub-tasks or require frequent communication between processes, making them challenging to parallelize efficiently (Valiev et al., 2010). Examples include wave-function-based *ab-initio* methods, such as CI, CC, and Multi-Configuration Self-Consistent Field (MCSCF) calculations (Hasanein and Evans, 1996). These methods often involve the manipulation of large matrices and the evaluation of high-dimensional arrays, making them computationally demanding and requiring sophisticated parallelization techniques to achieve optimal performance (Baumgartner et al., 2005).

Although frequently used quantum methods, such as Density Functional Theory (DFT) and semi-empirical methods, are more amenable to parallelization due to their relatively simpler mathematical formulations and reduced computational requirements (Andrade et al., 2015), they may not be optimal for calculations requiring a precise understanding of the electronic structure of the molecular system for elucidating the chemical phenomena at hand. Even these methods can face challenges in parallelizing specific aspects, such as evaluating long-range electron correlation or treating van der Waals interactions (Grimme et al., 2010).

The contrast between embarrassingly parallel tasks and challenging-to-parallelize quantum methods underscores the importance of developing advanced parallelization techniques and algorithms for various quantum chemistry applications. Researchers continue to explore approaches such as tensor decompositions, load balancing protocols, new hybrid MPI-OpenMP strategies, and computational hardware to overcome the limitations of current parallelization techniques for accelerating calculations (Götz et al., 2012). With ongoing developments in parallel computing, the potential to address computational challenges in quantum chemistry continues to expand, paving the way for more efficient and accurate calculations of complex molecular systems (Shao et al., 2015). Bearing this in mind, the following section provides some alternatives for calculating large molecules using CC methods.

## 4 Subdividing the correlation problem

In molecular orbital theory, the canonical set of molecular orbitals (MO) is typically generated through the linear combination of atom-centered basis functions, commonly Gaussian-type functions. These MOs are called canonical because they are the default MOs for a wave function obtained by diagonalizing the Fock matrix used in the SCF calculation of the HF method (Löwdin, 1955a; Löwdin, 1955b; Nesbet, 1955). Some coefficients in the linear combination can be zero or very small, which reduces computational complexity. This reduction occurs for several reasons.

- Symmetry: In many molecular systems, certain basis functions may not contribute to specific molecular orbitals due to symmetry constraints. The symmetry of the molecule can impose conditions on the coefficients of the linear combination, resulting in some of them being zero or negligible.
- Negligible overlap: When basis functions are centered on distant atoms, their overlap might be very small. In such cases, the contribution of these basis functions to the molecular orbitals can be negligible.
- Weak interactions in excited states: In the case of excited states, some molecular orbitals may have only weak interactions with each other. The contributions of these weakly interacting orbitals to the overall wave function can be minimal, leading to small coefficients in the linear combination.

These factors can simplify the representation of molecular orbitals and improve the efficiency of calculations by reducing the number of significant coefficients and matrix elements to consider (Boys, 1960; Simons and Nichols, 1997; Helgaker et al., 2000; Levine et al., 2009; Szabo and Ostlund, 2012).

Despite the possible reductions in canonical MOs, they can still lead to computational inefficiencies in large molecular systems. To address this issue, the set of canonical MOs can be transformed into an equally valid set of localized HF molecular orbitals through a unitary transformation that preserves orthonormality. These localized molecular orbitals (LMOs) not only correspond to chemically familiar concepts such as core orbitals on heavy atoms, bond orbitals, and lone pair orbitals, but can also be used to reduce the computational overhead in large molecular systems (Edmiston and Ruedenberg, 1963; Kleier et al., 1974; Pipek and Mezey, 1989; Subotnik and Head-Gordon, 2005; Herbert, 2019).

Transforming canonical MOs to LMOs suggest an intrinsic approach for studying large molecules. By studying a molecule as an assembly of smaller sub-molecules rather than as a whole, the conventional up-front computational overhead can be effectively reduced.

**FIGURE 1:**
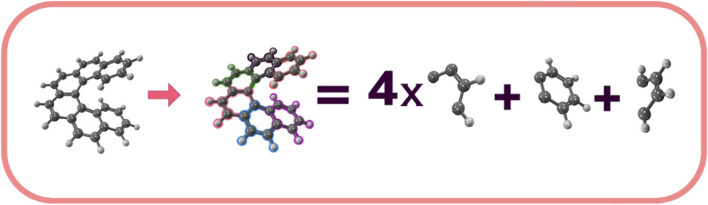
Decomposition of the [6] Helicene into C_4_H_4_, C_6_H_4_, and C_4_H_2_ fragments.

Following this molecular splitting idea, by dividing a large molecular system into smaller fragments and characterizing each fragment with wave-functions localized on specific molecular substructures, the electron correlation contributions for each fragment can be obtained through standard computation of matrix elements, allowing for the description of the entire molecular system by combining the wave-functions of all the fragments to form the final wave-function for the complete molecule. For instance, the [*n*]helicene molecule can serve as a practical example to illustrate the partitioning of a large molecular system into smaller fragments. This molecule can be partitioned into three primary molecular fragments, one with molecular formula C_4_H_4_, another with molecular formula C_6_H_4_, and *n* − 2 fragments with molecular formula C_4_H_2_. Consequently, the [6]Helicene (C_38_H_22_) molecule can be partitioned into one molecular segment with molecular formula C_4_H_4_, another with C_6_H_4_, and four with molecular formula C_4_H_2_, Figure 1 illustrates this partitioning. Under this partitioning scheme, the orbitals of each of the six fragments of the [6]Helicene molecule are confined to a linear combination of the basis set, possessing atomic functions centered on the C_4_H_4_, C_6_H_4_, and C_4_H_2_ nuclei, resulting in the comprehensive and final wave-function for the system. An efficient program design that takes advantage of this partition would distribute the workload for each fragment, establishing the orbitals for each fragment and computing the wave-functions and expectation values of each fragment in the initial phase. In the second phase, each fragment’s contribution would be added to obtain the complete description and total electronic energy of the entire molecule. This straightforward yet effective subdividing technique for computing the total energy of large systems embodies the core concept behind the Divide-Expand-Consolidate (DEC) scheme for correlated electron methods (Eriksen et al., 2015a; Ettenhuber et al., 2016; Kjærgaard et al., 2017a; Kjærgaard et al., 2017b; Barnes et al., 2019).

The DEC framework for correlated electrons involves dividing a large system into smaller fragments and obtaining the electron correlation contributions for each fragment through standard computation of matrix elements, similar to how it is done for small and intermediate systems. The following section outlines the DEC formalism for correlated wave-function methods, which enables the routine handling of molecular systems composed of over 200 atoms and more with a non-trivial number of electrons.

### 4.1 Localization of orbitals

A vital aspect of the DEC methodology is the localization of orbitals. Orbital localization forms the foundation for dividing large molecules into smaller fragments. Although the concept of localized orbitals is not new (Edmiston and Krauss, 1965), the literature contains a wide array of procedures aimed at localizing occupied and virtual orbitals as a means of electron-electron correlation calculations. Both wave function-based methods and fragmentation-based correlation approaches often generate localized orbitals through various localization procedures, such as those proposed by S.F. Boys (Boys, 1960) and J. Pipek and P.G. Mezey (Pipek and Mezey, 1989). However, these methods may be sensitive to the nature of molecular systems, which could limit their applicability. To address these limitations, advanced orbital localization techniques based on the central moment have been introduced (Jansík et al., 2011; Høyvik et al., 2012a; 2014). Combined with robust optimization strategies, these advanced techniques can provide more spatially local virtual orbitals even for traditionally delocalized systems (Høyvik et al., 2014; 2012a).

Localized orbitals that account for pair correlation effects have been pivotal in the development of electron-correlated methods (Löwdin, 1955a; Löwdin, 1955b; Edmiston and Ruedenberg, 1963; Davidson, 1972a; Davidson, 1972b; Pople et al., 1976; Surján, 1999). Pair natural orbitals (PNOs) are particularly useful for further compressing the virtual parameter space (Kapuy et al., 1983; Pulay, 1983; Pipek and Mezey, 1989; Saebø and Pulay, 1993; Neese et al., 2009; Yang et al., 2011; Yang et al., 2012). Constructed from (an approximation to) the MP2 correlation density matrix for each electron pair, PNOs have gained popularity in localized orbital CC methods (Pulay and Saebø, 1986; Hättig et al., 2006; Riplinger and Neese, 2013), such as the domain-based local PNO (DLPNO) CCSD(T) method (Neese et al., 2009; Wang et al., 2013). This method has been successfully applied to various computational chemistry applications (Riplinger et al., 2013; Riplinger and Neese, 2013; Sparta et al., 2017; Sharapa et al., 2019; Stoychev et al., 2021; Wang et al., 2022). Local CC methods relying on orbital-specific virtuals (OSVs) have also been developed, closely relating to PNOs (Kurashige et al., 2012; Yang et al., 2012; Schütz et al., 2013; Tew, 2019). However, PAOs can be non-orthogonal and redundant, complicating algorithmic expressions and posing conceptual challenges for molecular systems with degenerate states arising from symmetry and angular momentum coupling (Krause and Werner, 2012). Having a set of orthogonal and non-redundant localized virtual orbitals is beneficial for CC implementations and, in many cases, necessary for obtaining unambiguous results in molecular applications. Although, localized virtual orbitals obtained using advanced localization functions are more spatially local than PAOs (Høyvik et al., 2014; 2012a), despite their advantages, they might not always be the best choice for local correlated methods. The representation and selection of the optimal set of orbitals for local correlated methods remain active areas of research and continue to evolve.

### 4.2 Localized orbital-based correlation methods

Orbital localization is commonly utilized to express correlation calculations in a local basis, introducing approximations that reduce the computational complexity of a method in comparison to conventional implementations. Local correlation methods can be broadly classified into two categories, wave-function-based and fragment-based approximations.

Wave-function-based approximations focus on expressing the standard wave-function using a reduced parameter set. Such approximations commonly involve constraining the virtual excitation domain for each pair of occupied LMOs, while neglecting or approximating pair correlation contributions between well-separated occupied LMOs. For each LMO pair, a local correlation domain containing a subset of PAOs is assigned. PAOs, which form a non-orthogonal and redundant basis for the virtual orbital space, require adaptations to standard (canonical) algorithms to accommodate their unique properties. Notable examples of wave-function-based local approximations can be traced back to the work of Pulay and Sæbø (Pulay, 1983; Sæbø and Pulay, 1985; Pulay and Hamilton, 1988; Saebo and Pulay, 1988; Saebø and Pulay, 1993). These methods utilize occupied LMOs and PAOs for the virtual space, and have been further developed and expanded to gradients (Amos and Rice, 1989; Sæbø and Almlöf, 1989; Kirtman, 1995; Hampel and Werner, 1996; Russ and Crawford, 2004; Adler et al., 2009; Helgaker et al., 2012; Riplinger et al., 2013; Rolik et al., 2013; Menezes et al., 2016; Bistoni et al., 2017; Schwilk et al., 2017; Sitkiewicz et al., 2019; Saitow et al., 2022; Wang et al., 2022). In contrast, fragment-based approximations express the correlated method in amplitude equations and partition these amplitude equations into numerous small, typically independent, fragment calculations. Consequently, the energy is divided into fragment energy contributions, and the fragment energies are summed to yield the total energy. Examples of fragment-based approximations include the incremental CCSD(T) method (Friedrich and Dolg, 2009; Friedrich and Hänchen, 2013) and the local energy CCSD(T) method (Zhang and Grüneis, 2019; Altun et al., 2021).

Both wave function-based and fragment-based approximations have their advantages and drawbacks. Fragment-based approximations are better suited for modern multi-core architectures and have storage requirements independent of system size. In contrast, wave function-based approximations have storage requirements that grow with system size, which limits the size of the systems that can be treated. The choice of method depends on the specific application and the balance between computational cost and accuracy. As research in this area continues, further improvements and refinements of these methods are expected, enabling the treatment of larger and more complex molecular systems.

### 4.3 Breaking down correlation energy

In quantum chemistry theories, the concept of energy correlation, popularized by Löwdin (Löwdin, 1958), is the most prevalent perspective on the electron correlation problem. This description divides the exact total energy of a molecular system into the sum of the HF energy and a correlation contribution:

$$E_{\text{Total}}=E_{\text{HF}}+E_{\text{corr}}.$$ (1)

In general, for any correlated method, the relationship between the method’s total energy and the correlation energy can be expressed as:

$$E_{\text{Method}}=E_{\text{HF}}+E_{\text{corr}}.$$ (2)

In the case of the Møller-Plesset second-order perturbation theory (MP2) method, the energy expression in Eq. 2 becomes:

$$E_{\text{MP}2}=E_{\text{HF}}+E_{\text{corr}}^{\text{MP}2}.$$ (3)

For molecular systems, the MP2 correlation energy $E_{\text{corr}}^{\text{MP}2}$ can be expressed as:

$$E_{\text{corr}}^{\text{MP}2}=\underset{ij}{\sum }\underset{ab}{\sum }t_{ij}^{ab}\left(2g_{\mathrm{aibj}}-g_{\mathrm{biaj}}\right),$$ (4)

where *g*_*aibj*_ are the electron repulsion integrals (ERIs) using the Mulliken notation, and $t_{ij}^{ab}$ are the MP2 amplitudes.

Similarly, for the coupled cluster (CC) total energy (*E*_*CC*_), the correlation energy for a closed-shell molecular system can be obtained by:

$$E_{\text{corr}}^{\text{CC}}=\underset{ij}{\sum }\underset{ab}{\sum }\left(t_{ij}^{ab}+t_{i}^{a}t_{j}^{b}\right)\left(2g_{\mathrm{aibj}}-g_{\mathrm{biaj}}\right),$$ (5)

where $t_{i}^{a}$ and $t_{ij}^{ab}$ represent singles and doubles CC amplitudes. Indices *i*, *j*, … refer to occupied orbitals, while *a*, *b*, … refer to virtual orbitals. In the DEC coupled cluster (DEC-CC) framework, this correlation energy can be represented by a set of localized occupied and virtual HF MOs, which are determined and assigned to the atomic site nearest to the MO’s center of charge. Thus, the correlation energy for a given correlated method, *E*_corr_, becomes:

$$E_{\text{corr}}=\overset{N_{\text{frag}}}{\underset{P}{\sum }}\left[E_{P}+\overset{N_{\text{frag}}}{\underset{Q<P}{\sum }}\Delta E_{PQ}\right],$$ (6)

where *N*_frag_ is the number of atomic fragments the molecular system was divided into. For the CC method, the atomic fragment energy *E*_*P*_ and the pair fragment interaction energy Δ*E*_*PQ*_ are defined as follows:

$$E_{P}^{\text{CC}}=\underset{ij\in \underline{P}}{\sum }\underset{ab}{\sum }\left(t_{ij}^{ab}+t_{i}^{a}t_{j}^{b}\right)\left(2g_{\mathrm{aibj}}-g_{\mathrm{biaj}}\right),$$ (7)

$$\Delta E_{PQ}^{\text{CC}}=\underset{\begin{array}{c} i\in \underline{P}\;j\in \underline{Q} \end{array}}{\sum }\underset{ab}{\sum }\left(t_{ij}^{ab}+t_{i}^{a}t_{j}^{b}\right)\left(2g_{\mathrm{aibj}}-g_{\mathrm{biaj}}\right)+\underset{\begin{array}{c} i\in \underline{Q}\;j\in \underline{P} \end{array}}{\sum }\underset{ab}{\sum }\left(t_{ij}^{ab}+t_{i}^{a}t_{j}^{b}\right)\left(2g_{\mathrm{aibj}}-g_{\mathrm{biaj}}\right),$$ (8)

where the MOs are now assumed to be local, and $\underline{P}$ denotes the set of local occupied orbitals assigned to atomic site *P*.

It is important to note that the correlation energy expressions in Eqs 6–8 do not contain any approximations. Therefore, these equations, in principle, yield the same correlation energy corrections as the original expressions of the CC correlated methods. A crucial aspect of the DEC-CC approximation is dividing the calculation of the correlation energy of the entire molecular system into *N*_frag_ + 1/2 ⋅ *N*_frag_(*N*_frag_ − 1) independent molecular fragments. Computational savings arise when screening techniques are employed for each fragment calculation. In many instances, the locality of the MOs reduces the computational effort in the molecular calculation. Moreover, the integral *g*_*aibj*_ becomes negligible when the molecular orbital *ϕ*_*a*_ is spatially distant from *ϕ*_*i*_, allowing the summation over virtual orbitals in (7)–(8) to be limited. Consequently, only a subset of virtual orbitals, $\left[\bar{P}\right]$ , is significant for each fragment from the complete set of atomic site orbitals, *P*. A key advantage of the DEC framework is that these summation constraints in fragment energy calculations are determined in a black-box manner, enabling the definition of atomic fragment and pair fragment interaction CC energies as.

$$E_{P}=\underset{ij\in \underline{P}}{\sum }\underset{ab\in \left[\bar{P}\right]}{\sum }\left(t_{ij}^{ab}+t_{i}^{a}t_{j}^{b}\right)\left(2g_{\mathrm{aibj}}-g_{\mathrm{biaj}}\right),$$ (9)

$$\Delta E_{PQ}=\underset{\begin{array}{c} i\in \underline{P}\\ j\in \underline{Q} \end{array}}{\sum }\underset{ab\in \left[\bar{P}\right]\cup \left[\bar{Q}\right]}{\sum }\left(t_{ij}^{ab}+t_{i}^{a}t_{j}^{b}\right)\left(2g_{\mathrm{aibj}}-g_{\mathrm{biaj}}\right)+\underset{\begin{array}{c} i\in \underline{Q}\\ j\in \underline{P} \end{array}}{\sum }\underset{ab\in \left[\bar{P}\right]\cup \left[\bar{Q}\right]}{\sum }\left(t_{ij}^{ab}+t_{i}^{a}t_{j}^{b}\right)\left(2g_{\mathrm{aibj}}-g_{\mathrm{biaj}}\right),$$ (10)

where, for the pair fragment interaction energies, the set of virtual orbitals is chosen as the union of the atomic fragment spaces, which can be justified by a locality analysis of the results (Kristensen et al., 2011; Ettenhuber et al., 2016).

## 5 The Divide–Expand–Consolidate CCSD(T) framework

In the realm of computational quantum chemistry, the CCSD(T) model is often referred to as the *gold standard* for molecular calculations. Within the DEC framework, the CCSD(T) method is implemented as follows:

$$E^{\left(T\right)}=\overset{N_{\text{frag}}}{\underset{P}{\sum }}\left[E_{P}^{\left(T\right)}+\overset{N_{\text{frag}}}{\underset{Q<P}{\sum }}\Delta E_{PQ}^{\left(T\right)}\right]$$ (11)

with the corresponding equations.

$$E_{P}^{\left(T\right)}=2\underset{ij\in \underline{P}}{\sum }\;\underset{ab\in \left[\bar{P}\right]}{\sum }\left(2t_{ij}^{ab}-t_{ji}^{ab}\right)T_{ij}^{ab}+2\underset{i\in \underline{P}}{\sum }\;\underset{a\in \bar{P}}{\sum }t_{i}^{a}T_{i}^{a}$$ (12)

$$\Delta E_{PQ}^{\left(T\right)}=2(\underset{\begin{array}{c} i\in \underline{P}\\ j\in \underline{Q} \end{array}}{\sum }+\underset{\begin{array}{c} i\in \underline{Q}\\ j\in \underline{P} \end{array}}{\sum })\;\underset{ab\in \left[\bar{P}\right]\cup \left[\bar{Q}\right]}{\sum }\left(2t_{ij}^{ab}-t_{ji}^{ab}\right)T_{ij}^{ab}+2(\underset{\begin{array}{c} a\in \bar{P}\\ i\in \underline{Q} \end{array}}{\sum }+\underset{\begin{array}{c} a\in \bar{Q}\\ i\in \underline{P} \end{array}}{\sum })t_{i}^{a}T_{i}^{a}.$$ (13)

In these expressions, the intermediate terms $T_{ij}^{ab}$ and $T_{j}^{a}$ can be defined as follows.

$$T_{ij}^{ab}=\underset{cd\in \left[\bar{P}\right]}{\sum }\underset{k\in \left[\underline{P}\right]}{\sum }\left(t_{\mathrm{ijk}}^{\mathrm{acd}}L_{\mathrm{bckd}}-t_{\mathrm{kji}}^{\mathrm{acd}}g_{\mathrm{kdbc}}\right)-\underset{c\in \left[\bar{P}\right]}{\sum }\underset{kl\in \left[\underline{P}\right]}{\sum }\left(t_{\mathrm{ikl}}^{\mathrm{abc}}L_{\mathrm{kjlc}}-t_{\mathrm{lki}}^{\mathrm{abc}}g_{\mathrm{kjlc}}\right)$$ (14a)

$$T_{i}^{a}=\underset{cd\in \left[\bar{P}\right]}{\sum }\underset{kl\in \left[\underline{P}\right]}{\sum }\left(t_{\mathrm{ikl}}^{\mathrm{acd}}-t_{\mathrm{lki}}^{\mathrm{acd}}\right)L_{\mathrm{kcld}}.$$ (14b)

Here, $\left[\underline{P}\right]$ represents the set of occupied orbitals assigned to atomic sites near center *P*, analogous to the virtual spaces. The triples amplitudes $t_{\mathrm{ijk}}^{\mathrm{abc}}$ are derived from the CCSD doubles amplitudes, and *L*_*aibj*_ = 2*g*_*aibj*_ − *g*_*biaj*_. This implementation of CCSD(T) necessitates an additional *o* ^3^ *v* ^4^ scaling step compared to a conventional CCSD(T) implementation, as the standard CCSD(T) method cannot be easily partitioned into atomic fragment energy contributions due to the (T) corrections (Raghavachari et al., 1989). Nevertheless, with this implementation, the CCSD(T) method can be partitioned analogously to the standard CC correlation energy (Eriksen et al., 2015a).

The energy partitioning presented in Eqs 9, 10 defines what is known as the occupied partitioning scheme. However, DEC utilizes both local occupied and local virtual orbitals, and as a result, virtual and Lagrangian partitioning schemes are also formulated (Høyvik et al., 2012a). These partitioning schemes not only provide independent paths for evaluating correlation energy, maintain comparable error control, and yield reliable results, but they also exhibit distinct characteristics. The virtual and Lagrangian partitioning schemes tend to generate larger fragments in practice, yet they still enable error estimation in DEC calculations. In contrast, the Lagrangian scheme offers some advantages over the occupied and virtual schemes due to its variational nature, which leads to errors in amplitudes and multipliers being roughly proportional to the square root of the fragment optimization threshold (FOT). Additionally, the Lagrangian scheme delivers a more balanced treatment of both occupied and virtual spaces. Although virtual orbitals are generally less localized than occupied orbitals (Høyvik and Jørgensen, 2016), resulting in larger fragments within the virtual and Lagrangian schemes compared to the preferred occupied scheme, DEC calculations of molecular gradients necessitate the use of the virtual partitioning scheme. (Kristensen et al., 2012a; Bykov et al., 2016).

### 5.1 Atomic fragment optimization

As a continuation of the discussion on partitioning the correlation energy into atomic fragment and pair fragment interaction energies, this section focuses on optimizing the occupied and virtual orbital spaces $\left[\underline{P}\right]$ and $\left[\bar{P}\right]$ for atomic fragment *P*. The error associated with this optimization is dictated by the FOT used to obtain the fragment energy *E*_*P*_.

The atomic fragment energy *E*_*P*_ in Eq. 9 is determined from the energy orbital space (EOS), $P_{\text{EOS}}\equiv \underline{P}\cup \left[\bar{P}\right]$ . The EOS represents the orbital space which ensures accurate corrlation energy. However, due to the coupling between the CC amplitudes, solving the CC amplitude equation in *P*_EOS_ is not feasible (Eriksen et al., 2015a; Ettenhuber et al., 2016). Instead, the coupling can be accounted for by solving the CC amplitude equations in an extended orbital space, the amplitude orbital space (AOS), $P_{\text{AOS}}\equiv \left[\underline{P}\right]\cup \left[\bar{P}\right]$ (Eriksen et al., 2015a). It is important to note that the occupied orbitals in the EOS $\left(i\in \underline{P}\right)$ are fixed by the orbital assignment, and the virtual orbital space is identical for both EOS and AOS. Assuming $\left[\underline{P}\right]$ and $\left[\bar{P}\right]$ are known, the atomic fragment energy *E*_*P*_ can be calculated as follows.

1. Solve the CC amplitude equations in *P*_AOS_.
2. Extract the CC amplitudes from *P*_AOS_ to *P*_EOS_.
3. Calculate the two-electron integrals in *P*_EOS_.
4. Use the CC amplitudes and integrals in *P*_EOS_ to calculate the atomic fragment energy as in Eq 9.

In DEC, the strategy to determine the spaces $\left[\underline{P}\right]$ and $\left[\bar{P}\right]$ that yield atomic fragment energies with FOT accuracy consists of two steps: fragment expansion followed by fragment reduction (Ettenhuber et al., 2016). In the fragment expansion step, a priority list $l_{r}^{P}$ is generated to describe the importance of each local orbital for the fragment energy *E*_*P*_, utilizing the distance between the center of charge of a given orbital and the atomic site *P* due to the locality of correlation effects (Høyvik and Jørgensen, 2016). However, alternative lists based on numerical overlap of orbitals or Fock matrix elements have also been tested with similar results (Ettenhuber et al., 2016). The process begins by selecting an initial space ( $\left[\underline{P}\right]$ and $\left[\bar{P}\right]$ ) from the priority list, calculating the fragment energy as previously described, then expanding the orbital spaces based on the priority list, and subsequently obtaining an improved fragment energy. This procedure is repeated until the difference between the last two fragment energies falls below the FOT. The fragment reduction step involves a binary search to remove orbitals without introducing errors larger than the FOT in the atomic fragment energy (Ettenhuber et al., 2016). This reduces the size of the AOS for atomic fragments, leading to significant computational savings for pair fragments.

The error control of the atomic fragment optimization comes with overhead, and improving the fragment optimization procedure is an ongoing research direction in optimizing the DEC scheme (Riplinger and Neese, 2013; Ettenhuber et al., 2016). One possible approach to reducing the overhead is to explore more efficient algorithms or heuristics that can guide the optimization process and reduce the number of calculations needed to reach the desired FOT accuracy.

Additionally, for DEC-CCSD or DEC-CCSD(T) calculations, fragment optimization can be performed at a lower level of theory, such as DEC-MP2 (Riplinger and Neese, 2013a; Ettenhuber et al., 2016). This approach can lead to considerable computational savings without significantly compromising the accuracy of the final results. However, it is crucial to validate the appropriateness of using a lower level of theory for the specific system being studied, as some systems might require higher levels of theory for accurate predictions.

The locality of electron correlation is system-dependent, and the goal of the fragment optimization procedure is to obtain a method that provides the same recovery of the correlation energy for all systems, independently of the complexity of the electronic structure (Høyvik and Jørgensen, 2016). The fragment spaces tend to be larger for systems characterized by a delocalized electronic-structure, such as graphene, than for systems containing only non-conjugated covalent bonds. In particular, for systems with a delocalized electronic-structure, it is important to use the most advanced orbital localization functions, such as the squared fourth moment localization function (Høyvik et al., 2012a), as these localization functions can generate localized sets of orbitals that are minimally system-dependent (Høyvik and Jørgensen, 2016).

Furthermore, incorporating machine learning techniques into the fragment optimization procedure may offer a promising avenue for future research (Westermayr et al., 2021). By training machine learning models on existing datasets of molecular systems, it may be possible to predict optimal fragment spaces or guide the optimization process more efficiently. This approach could potentially reduce the computational cost associated with the optimization procedure while maintaining the desired level of accuracy.

### 5.2 DEC amplitudes and the transformation of the basis

In the fragment energy calculations of the DEC scheme, the CC amplitude equations must be solved in the AOS. To achieve this, the set of local orbitals is transformed into a pseudo-canonical basis by diagonalizing the local Fock matrix blocks *F*_*ij*_ $\left(ij\in \left[\underline{P}\right]\right)$ and *F*_*ab*_ $\left(ab\in \left[\bar{P}\right]\right)$ . The pseudo-canonical basis is traditionally denoted using capital letters *I*, *J*, *A*, *B*.

The CC amplitude equations are better conditioned in the pseudo-canonical basis, and the MP2 amplitudes (Kristensen et al., 2011; Høyvik et al., 2012b) and (T) intermediates (Ziółkowski et al., 2010; Eriksen et al., 2015a) can be obtained non-iteratively using standard canonical CC algorithms. After solving the amplitude equations in the AOS, the amplitudes must be transformed back to the local basis $\left(t_{IJ}^{AB}\to t_{ij}^{ab}\right)$ in order to extract the EOS amplitudes and calculate the fragment energy. A similar operation is performed for the (T) intermediates ( $T_{ij}^{ab}$ and $T_{i}^{a}$ in equation (14)).

While the pseudo-canonical basis is utilized throughout the code implementation, the transformation to the local basis is model-dependent. It is worth noting that when the energy is evaluated using the occupied partitioning scheme in Eqs. 9, 10, transforming the virtual orbitals to the local basis is not necessary. Moreover, in the case of the Laplace-transformed variation of the method DEC-RI-MP2 (i.e., the DEC-LT-RIMP2 method), the amplitudes are directly obtained in the local basis (Bykov et al., 2016; Bykov and Kjaergaard, 2017).

### 5.3 Pair fragment calculations

The linear scaling of the DEC scheme is achieved only when the number of pair fragments scales linearly with the system size. Dispersion interactions cause the pair fragment interaction energy Δ*E*_*PQ*_ to decay following the $R_{PQ}^{-6}$ pattern with the distance *R*_*PQ*_ between atomic sites *P* and *Q*. By employing a real-space cutoff *R*_screen_, pairs with a distance exceeding the distance screening threshold (e.g., *R*_*PQ*_ > *R*_screen_) can be screened, leading to a linear-scaling algorithm (Ziółkowski et al., 2010; Kristensen et al., 2011; Kristensen et al., 2012a; Høyvik et al., 2012b; Kristensen et al., 2012b; Jakobsen et al., 2013; Olsen et al., 2015). However, this strategy presents two challenges: (i) the calculated number of pair fragment interaction energies becomes independent of the FOT, requiring adjustments to both the FOT and *R*_screen_ in order to converge to the standard CC correlation energy, and (ii) the pair fragment interaction energies for a specific pair distance can span several orders of magnitude indicating that numerous pairs included within the distance screening threshold are less significant than some of the pairs excluded. To address these challenges, a pair screening strategy based on estimating the pair fragment interaction energies can be considered. This strategy is introduced by rewriting the correlation energy (Eq. 6) as a sum of effective atomic fragment energies *ϵ*_*P*_,

$$E_{\mathrm{corr}}=\overset{N_{\text{frag}}}{\underset{P}{\sum }}\epsilon_{P}.$$ (15)

The effective atomic fragment energy for fragment *P* is the sum of the atomic fragment energy *E*_*P*_ and the average pair fragment interaction energy $E_{P}^{av}$ . The latter term describes the interaction between the atomic site *P* and the other atomic sites,

$$\epsilon_{P}=E_{P}+E_{P}^{av},$$ (16)

with

$$E_{P}^{av}=\frac{1}{2}\underset{Q\neq P}{\sum }\Delta E_{PQ}.$$ (17)

This process of partitioning a system into fragments is not exclusive to DEC. Since it is also employed in other fragment-based quantum chemistry methods (Wang et al., 2014; Fedorov, 2017).

In order to accurately determine the average pair fragment interaction energy to the FOT accuracy, a pair fragment screening technique is employed. This technique involves using minimal orbital spaces to calculate pair energy estimates at the MP2 level, based on a priority list $l_{r}^{P}$ , such that the estimates recover a significant portion (usually 80–95%) of the *exact* MP2 pair fragment interaction energies, while requiring much less computational resources. To ensure efficiency, a conservative real-space cutoff of *R*_screen_ = 30Å ensures a linear-scaling number of pair energy estimates.

Once the pair energy estimates have been obtained, the screening proceeds by following this specific strategy.

1. Order all pair energy estimates associated with a *N*_frag_ number of atomic sites in the molecule within a given *P* fragment,

$$|\Delta E_{P1}^{\text{esti}}|\leq |\Delta E_{P2}^{\text{esti}}|\cdots \leq |\Delta E_{PN_{\text{frag}}}^{\text{esti}}|,$$ (18)

2. Sum up the estimated contributions in the list, starting with the smallest values until it adds up to the FOT,

$$\underset{I_{P}}{\max}\left(\frac{1}{2}\overset{I_{P}}{\underset{Q=1}{\sum }}|\Delta E_{PQ}^{\text{esti}}|\right)\leq \text{FOT}$$ (19)

3. All pairs $\Delta E_{PQ}^{\text{esti}}$ in the ordered list for which *Q* ≤ *I*_*P*_ are then screened away and not calculated at the target CC level.
4. Repeat Steps 1-3 for all atomic sites.

This procedure, combined with fragment optimization and basis transformation, serves as the core of the DEC framework. It facilitates the breakdown of large molecular systems into smaller, more manageable fragments that can be treated independently. Consequently, it enables a series of single fragment and pair fragment calculations, which concentrate on specific sections of the system, and offers the potential to take advantage of tensor hypercontraction concepts and linear-scaling approaches.

### 5.4 Error estimates

As emphasized earlier, a crucial aspect of practicing computational quantum chemistry is to recognize and appreciate the accuracy limitations inherent to the different approaches that make up the computational molecular method.

In connection with the approximations found in the DEC framework, it is important to recognize the three primary assumptions that greatly impact the calculation’s accuracy: (i) the fragment expansion procedure, which is presumed to converge with an error deemed negligible relative to the FOT, (ii) the pair energy estimates and their ability to deliver a qualitative approximation of the actual pair fragment interaction energies, confirming that the relevant pair fragment interaction energies are assessed, and (iii) the errors in the ultimate pair fragment interaction energies *E*_*PQ*_ being minimal in comparison to the FOT.

The FOT is a crucial aspect of the DEC framework, as it directly governs the correlation energy error and implicitly manages errors in the correlated density matrix and molecular gradient. The DEC framework has been purposefully designed to control the error in atomic fragment energy *E*_*P*_ and average pair fragment interaction energy $E_{P}^{av}$ according to the FOT. This is achieved by determining the AOS for each atomic fragment in a DEC calculation, ensuring precision dictated by the FOT through a black-box like algorithm. The AOS is initially defined with only the nearest neighbor atoms to *P* and expands layer by layer, assessing the energy contribution from each individual orbital in the AOS. It is then examined whether single orbitals can be removed from the AOS without affecting the calculation’s precision, allowing the atomic fragment energies to be determined within the FOT tolerance.

DEC calculations derive properties from the sum of contributions from atomic fragment and pair fragment calculations, determined up to the FOT tolerance. An analysis of numerical experiments has revealed that the accuracy of a DEC calculation is influenced by a correlation error, which scales with *N*_fragments_ and is dependent on the number of non-Hydrogen atoms in the system, such that

$$\delta E_{\text{corr}}\approx 2N_{\text{frag}}.\text{FOT}$$ (20)

The error of a DEC calculation compared to a conventional calculation depends on the FOT for size-intensive properties but relies on both the FOT and system size for size-extensive properties. For example, the percentage of correlation energy recovered for a given FOT is independent of the system size, while the absolute error in the correlation energy increases linearly with the system size. Similarly, the error at a specific point in space of the correlated density or electrostatic potential depends solely on the FOT, irrespective of the system size.

On the other hand, the error control of the DEC method has been validated through theoretical analyses and numerical results for small and medium-sized molecules. For larger molecules, internal consistency checks can be used to estimate errors in the calculated DEC correlation energy and electron density, revealing that DEC errors for size-intensive properties remain consistent across different system sizes, while errors for size-extensive properties increase with system size.

DEC calculations are capable of recovering over 99% of the correlation energy if a sufficiently stringent FOT is employed, Table 1.

**TABLE 1:** Percentage of correlation recovered as a function of the FOT.

FOT	Δ_DEC_ (%)
10^–3^	98.2
10^–4^	99.8
10^–5^	99.985
10^–6^	99.998

Although FOT can assure a reduction in error compared to the method’s conventional counterpart, it does not guarantee the validity of the calculation’s accuracy for the system being studied. This point is related to a minor alteration of the earlier reflective question:

What is the maximum number of atoms for which electronic energy calculations and quantum mechanical methods remain meaningful and do not lose their significance?

### 5.5 The Divide–Expand–Consolidate parallelization

The DEC algorithm’s attractiveness for high-performance computing is due in part to its core procedures: fragment optimization, local energy calculation, and pair fragment screening. These processes enable the DEC framework to divide large molecular systems into independent fragments and taking advantage of tensor hypercontraction concepts (like resolution-of-identity) and other linear-scaling procedures and optimization on the fragment level. The method has been applied to study sizable systems, such as supramolecular wires with up to 40 monomers of 1-aza-adamantane-trione (AAT) molecules, encompassing 2,440 atoms and 24,440 basis functions (Kjærgaard et al., 2017a; Kjærgaard et al., 2017b).

Three levels of parallelization using a multi-threaded OpenMP and MPI implementation exist within the DEC algorithm: coarse, medium, and fine-grained. Coarse-grained parallelization leverages the independent nature of fragment energy calculations, allowing them to be executed in parallel on separate computing units (usually a user defined team of compute nodes). Meanwhile, medium and fine-grained parallelization focus on distributing the solution of the CC amplitude equations for each molecular fragment across multiple compute nodes within computing unit and individual threads on a particular node.

At the coarse-grained level, available compute units are divided into groups under a global work manager that directs the DEC calculation through a set of local managers. These local managers each command a group of workers to execute individual fragment calculations. The global manager generates an ordered job list based on the workload of each job, which represents an atomic or pair fragment interaction energy calculation. The global manager communicates the required information for each fragment energy calculation to local groups, starting with the largest fragments. As local groups become available, the global manager dynamically distributes the remaining fragment calculations in the job list. The size of the local group is also dynamic, splitting if the workload is insufficient to span across the local group, with two new jobs assigned to the new groups. This combination of dynamic job distribution and dynamic group size adjustment minimizes global and local communication losses, ensuring optimal performance on large parallel machines. Finally, the global manager calculates the correlation energy based on Eq 6.

The DEC scheme has been used on large compute clusters and supercomputers like Titan (Kjærgaard et al., 2017a; Kjærgaard et al., 2017b) and Summit (Luo et al., 2020) at Oak Ridge National Laboratory, as well as on GPUs (Bykov and Kjaergaard, 2017). Several methods have been implemented using the DEC strategy, including DEC resolution-of-the-identity MP2 (Bykov and Kjaergaard, 2017) with gradients (Bykov et al., 2016), densities (Kristensen et al., 2012a), Laplace–transformed (Kjærgaard, 2017), and Explicitly–correlated (Wang et al., 2016) excitation energies using local framework (LoFEx) (Baudin and Kristensen, 2016), CCSD (Baudin et al., 2017); CCSD(T) (Eriksen et al., 2015a); and multi–layer DEC (Barnes et al., 2019).

### 5.6 Demonstrative calculations

**FIGURE 2:**
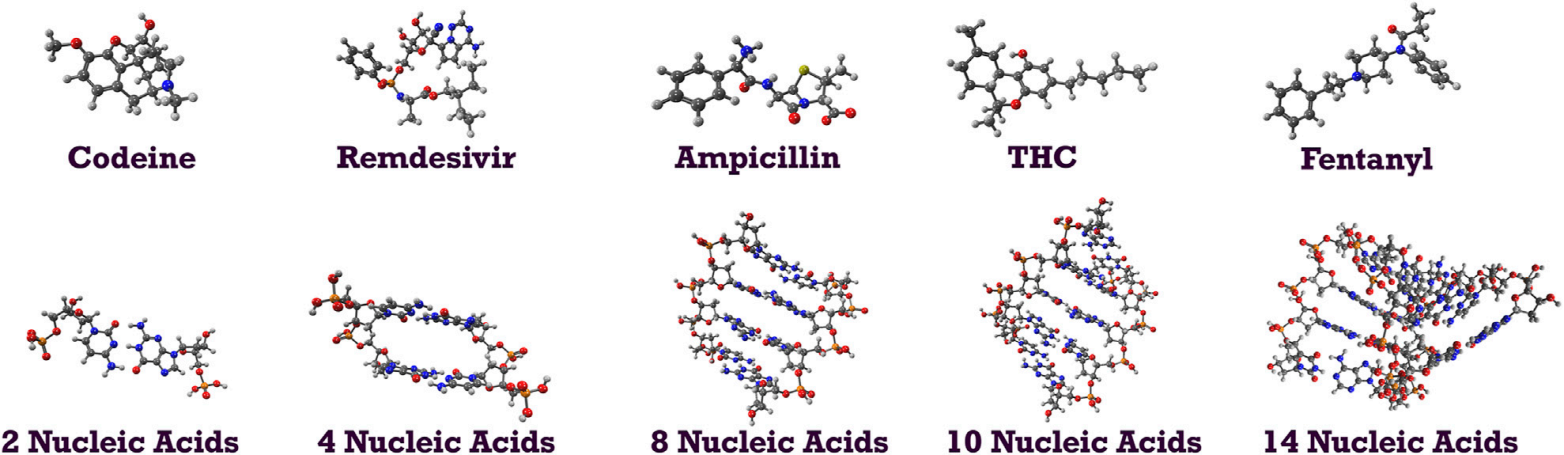
Molecular structures of codeine (pain reliever), remdesivir (antiviral), ampicillin (antibiotic), THC (principal psychoactive constituent of cannabis), fentanyl (pain medication); molecular structures of B-DNA fragments with 2, 4, 8, 10, and 14 nucleic acids.

**FIGURE 3:**
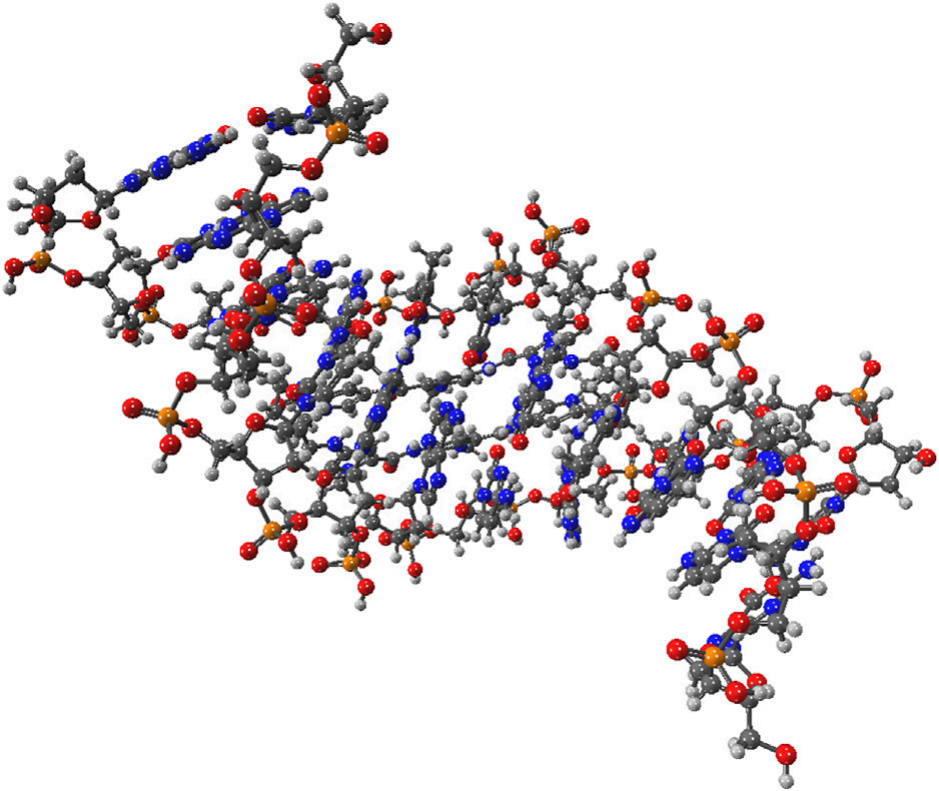
Molecular structure of the protonated Cyclic Polyamide-DNA Complex.

To demonstrate the capability of the DEC methodology for incorporating correlated techniques, we applied it to simulate 11 biologically and pharmacologically significant molecules at the MP2 level, utilizing the resolution of identity (RI) approximation in conjunction with the cc-pVDZ basis set (Dunning Jr, 1989). The optimized molecular geometries were obtained with the Berny algorithm available in the Gaussian 16 suite of programs (Frisch et al., 2021). Initial guess geometries for codeine, remdesivir, ampicillin, tetrahydrocannabinol (THC), and fentanyl were computed in the gas phase using Hartree-Fock (HF) and the 6–311++G (d,p) basis set (Krishnan et al., 1980; Frisch et al., 1984). These structures were subsequently optimized in water and pentylethanoate with the Polarizable Continuum Model (PCM) to simulate the aqueous and lipidic effects within a biological cellular system. Initial structures for protonated deoxyribonucleic acid (DNA) fragments were optimized with HF/STO-3G (Hehre et al., 1969; Collins et al., 1976) in both gas and aqueous phases. These initial geometries were later refined using the BP86 functional with Orca (Neese et al., 2020) and the cc-pVDZ basis set. Figure 2 illustrates the molecular structures of five protonated canonical right-handed DNA helix (B-DNA) fragments with two (B-DNA (2)), four (B-DNA (4)), eight (B-DNA (8)), and ten (B-DNA (10)) nucleic acids, as well as five common drugs (pain relievers, an antiviral, an antibiotic, and a cannabinoid). Additionally, the molecular structure reported by Q. Zhang et al. for a protonated Cyclic Polyamide-DNA Complex, FDNA (Zhang et al., 2004), was considered in this study and computed with the cc-pVDZ basis set (see Figure 3).

DEC framework offers a significant advantage in its adaptability to established techniques for accelerating molecular calculations. One such technique is RI for reducing four-index integrals, which offers substantial computational savings for DEC calculations. The RI approximation can be straightforwardly applied on the fragment level for any CC level of excitation.

Table 2 displays the HF and RIMP2 total energy values for the 11 computed molecules. The results demonstrate the accuracy of the DEC framework for modeling large and complex systems, as well as its effectiveness in reducing computational cost. Table 3 presents the CCSD total energies for three representative molecules. These results further showcase the adaptability of the DEC framework to more advanced correlated methods and its ability to provide accurate results for large and complex systems. The flexible nature of the DEC framework makes it amenable to the integration of various correlated methods for the computation of total energies and molecular properties.

**TABLE 2:** DEC-RIMP2 total energy for 11 biologically relevant molecules, all values are in a.u.

Molecule	Hartree-Fock energy	RIMP2 total energy
Codeine	−972.862701	−975.992852
Remdesivir	−2,309.345791	−2,315.468663
Ampicillin	−1477.391759	−1480.860488
THC	−962.501681	−965.745358
Fentanyl	−1033.262311	−1036.726093
B-DNA (2)	−2,901.318601	−2,907.974428
B-DNA (4)	−5650.439019	−5663.251418
B-DNA (8)	−11148.833671	−11174.064299
B-DNA (10)	−13882.030042	−13913.440352
B-DNA (14)	−19348.404412	−19392.241663
FDNA	−32403.080000	−32480.965782

**TABLE 3:** DEC-CCSD total energies for three representative molecules, all values in a.u.

Molecule	Hartree-Fock energy	CCSD total energy
Codeine	−972.862700	−976.107748
Fentanyl	−1033.262311	−1036.877810
THC	−962.501681	−965.914124

The DEC approximation is particularly useful if time-to-solution is to be optimized. Due to Embarrassingly parallel nature of the DEC the time-to-solution can be brought down practically to any number provided a computing resources are available. Obviously, this comes at the cost of much higher count of floating point operations (FLOPs). Thus for small and medium size systems it would be more beneficial to use canonical implementation or approximations able to be optimized for the optimal FLOPs count.

The DEC framework presents a flexible and adaptable strategy for modeling intricate and large molecular systems without compromising precision and computational efficiency. By leveraging well established techniques such as the RI approximation and integrating advanced correlated methods, the DEC framework empowers computational chemistry in HPC with a powerful tool. Although, the DEC framework provides a versatile and robust black-box like approach to addressing the challenges of contemporary molecular modeling, there are still several obstacles that must be overcome to establish it as an essential tool in the realm of computational chemistry.

### 5.7 Challenges and opportunities

Although the DEC framework combined with electron correlation methods may appear attractive for computing large systems, practitioners in computational chemistry should be mindful of the applicability and limitations of each method. It is crucial to remmmber that a given model is only useful within a specific range of applications, and its validity may not extend to all types of molecular systems or sizes. In this regard, the DEC framework with MP2 and CC methods presents a vulnerability; while MP2 and CC methods have demonstrated their accuracy and effectiveness for small to medium molecular systems, their systematic validation for large systems remains to be conducted.

Moreover, many atomic and molecular processes necessary for experimental applications are described by measurements inferred from energy differences. A single total energy of a molecular system is not chemically relevant unless the energies for the initial and final states, accounting for the change in energy during the chemical process, are available. Additionally, it has been established that an accuracy of 1 kcal/mol is necessary for quantum mechanical simulations to make reliable predictions for thermochemical properties, chemical kinetics, reactivity profiling, and chemical transformations. The use of DEC or other fragmentation approaches for molecular fragments can result in the recovery of correlation energy exceeding 1 kcal/mol for large molecules. This raises concerns about the validity of the electronic energy error propagation in large systems. When the fragments computed have a contribution to the total energy larger than the accepted error of 1 kcal/mol, it becomes challenging to discuss the accuracy of the correlated total energy.

Moreover, for large molecular systems, electron-electron effects and electronic energy become less important due to the contribution of vibrational effects. Therefore, practitioners should consider the largest number of atoms for which it is still reasonable to compute solutions of the Schrödinger equation. Although a systematic evaluation of the upper limit size for quantum chemistry methods is yet to be conducted, it is well-known that for large molecular systems, the transition from a quantum mechanical domain to a statistical mechanical one occurs. Depending on the system size, one should evaluate whether to use approaches from quantum mechanics, statistical mechanics, fluid mechanics, or thermodynamics.

Nevertheless, correlated electron methods such as CI, CC, and DEC CC can play a crucial role in the development and training of ML and AI chemical applications, where electronic structure elucidation becomes secondary and pragmatic insights are paramount for task prioritization. The DEC framework, in particular, could be a natural choice for ML chemistry models with architectures based on diffusion, active learning, generative adversarial models, normalizing flows, and transformers.

For large systems, properties depending on a correct description of electronic behavior are often localized to a specific site in the molecule or molecular system. Accounting for all electron-electron correlations may be computationally wasteful due to the small contributions from electrons far from the molecular site where the electronic process occurs. Furthermore, accessing many final states needed to describe chemical transformations can be challenging due to variational collapse, symmetry breaking, spin contamination, and the consideration of multiple configurations. Thus, direct methods for computing localized electron-electron properties should be a more effective way to tackle electron-driven phenomena in large molecules. Quantum chemistry methods that directly compute the desired property of the molecule, such as those based on Green’s functions, equation of motion and response theories, are particularly important for large molecules. In the following section, the application of one such method in the framework of the CC theory will be explored.

## 6 Cluster perturbation theory

An alternative method to the DEC-CC model and other localized *ab-initio* approaches that can be efficiently adapted for use on modern supercomputers to enable CC quality calculations on large molecular systems is the recently developed Cluster Perturbation theory (CP) (Baudin et al., 2019; Pawłowski et al., 2019a; Pawłowski et al., 2019b; Pawłowski et al., 2019c; Pawłowski et al., 2019d; Hillers-Bendtsen et al., 2022; Høyer et al., 2022; Olsen et al., 2022). CP theory offers a hybrid approach that combines CC theory and the Møller-Plesset (Møller and Plesset, 1934) partitioning of the wave-function. This unique combination helps overcome the limitations of the individual models and offers a more comprehensive and accurate approach to molecular calculations (Bartlett and Silver, 1974; Binkley and Pople, 1975; Pople et al., 1976; Bartlett and Shavitt, 1977; Krishnan and Pople, 1978; Raghavachari et al., 1989; Eriksen et al., 2014; 2015b; Eriksen et al., 2015c; Kristensen et al., 2016).

CP theory introduces a new class of perturbation models that rely on a correlated zeroth-order state, which can be truncated at any excitation level, thereby avoiding high excitation levels that have a negligible effect on molecular properties. Perturbation series for both energy and molecular properties, including excitation energies, can be determined by calculating small perturbation corrections, which are obtained by taking the difference between the energy and molecular property for the CC parent and CC target states.

Compared to coupled cluster perturbation theory (CCPT) energy and CCPT Lagrangian series, CP theory exhibits a faster convergence and yields superior results. It allows for the calculation of perturbation series for both ground-state energy and excitation energies, treating the CC parent state Jacobian as a zeroth-order contribution. Excitation energies in CP theory can be determined using either response function theory or equation-of-motion coupled cluster (EOM-CC) theory.

CP theory’s unique pathway that connects the determination of the CC parent state and the CC target state makes it possible to determine CP series for both energy and molecular properties. Moreover, its incorporation of only one additional excitation level typically results in a more robust perturbation series and, hence, better convergence. Furthermore, non-iterative and easily parallelizable formulations can be derived within CP theory and some the targeted model is a higher-level CC model rather than a full configuration interaction (FCI) solution, the derived corrections remain reasonably small.

A brief introduction to CP theory for electronic structure calculations, specifically for excitation energies, is presented in the following section. Additionally, the development of massively parallel implementations of CP theory and some numerical illustrations are discussed. For a comprehensive understanding of CP theory, interested readers may refer to (Pawłowski et al., 2019a,b; Baudin et al., 2019; Pawłowski et al., 2019c,d; Høyer et al., 2022; Olsen et al., 2022; Hillers-Bendtsen et al., 2022).

### 6.1 Standard CC theory for excitation energies

In the standard CC theory, the coupled cluster (CC) wave function (Čížek, 1966) is written as

$$|CC\rangle =e^{T}|HF\rangle ,$$ (21)

where |HF⟩ is the Hartree-Fock reference state. Furthermore, in the above expression the cluster operator reads

$$T=\underset{i}{\sum }\underset{\mu_{i}}{\sum }t_{\mu_{i}}\theta_{\mu_{i}},$$ (22)

where the cluster amplitude is denoted $t_{\mu_{i}}$ , and it is associated with the many-body excitation operator $\theta_{\mu_{i}}$ which, when applied to the Hartree-Fock reference state, generates an *excited* determinant: $|\mu_{i}\rangle =\theta_{\mu_{i}}|HF\rangle$ . Thus, the energy may be determined as

$$E_{0}=\left\langle HF|e^{-T}H_{0}e^{T}|HF\right\rangle =\left\langle HF|H_{0}^{T}|HF\right\rangle .$$ (23)

In this energy expression, the electronic Schrödinger equation has been multiplied from the left with *e* ^−*T*^ and then projected against the Hartree-Fock state. Consequently, the corresponding amplitude equations are given as

$$\left\langle \mu_{i}|e^{-T}H_{0}e^{T}|HF\right\rangle =\left\langle \mu_{i}|H_{0}^{T}|HF\right\rangle =0.$$ (24)

The similarity transformed Hamiltonian has been introduced,

$$H_{0}^{T}=e^{-T}H_{0}e^{T}.$$ (25)

Based on linear response theory, the CC excitation energies may be obtained as eigenvalues of the CC Jacobian, ***J*** (Harris, 1977; Dalgaard and Monkhorst, 1983), such that,

$$JR_{x}=\omega_{x}R_{x},$$ (26)

$$L_{x}J=L_{x}\omega_{x},$$ (27)

$$L_{x}R_{y}=\delta_{xy},$$ (28)

The CC Jacobian,

$$J_{\mu_{i}\nu_{j}}=\left\langle \mu_{i}|\left[H_{0}^{T},\theta_{\nu_{j}}\right]|HF\right\rangle ,$$ (29)

is non-Hermitian and therefore the left and right egienvectors, ***L***_*x*_ and ***R***_*x*_ (which represent the excited-state wave function) are not Hermitian conjugates of each other. The Jacobian formulation of CP theory defines a numerical problem that can be solved using a block Davidson eigensolver, where the eigenvalues are determined by an iterative procedure starting from the lowest eigenvalue.

### 6.2 Cluster perturbation theory for excitation energies

For excitation energies, the CC target excitation space, 1 ≤ *i* ≤ *t*, is divided into two, the parent excitation space and the auxiliary excitation space. The parent excitation space comprises the excitations from 1 ≤ *i* ≤ *p*, whereas the auxiliary space includes the excitations from *p* < *i* ≤ *t*.

The CC parent state wave–function is defined as^1^

|CC*〉=eT*|HF〉. (30)

This wave–function is defined by a truncated cluster operator which covers the excitations in the parent space.

T*=∑i=1pTi*, (31)

Ti*=∑μitμi*θμi. (32)

Then, the corresponding energy and amplitude equations for the parent state read

E0*=⟨HF|H0*T|HF⟩, (33)

⟨μi|H0T*|HF⟩=0,1≤i≤p. (34)

Subsequently, the excitation energies are obtained from the eigenvalue problem involving the Jacobian of the parent space which is denoted by the left superscript *P*,

JPRx*=ωx*Rx*, (35)

Lx*JP=Lx*ωx*, (36)

JμiνjP=⟨μi|H0T*,θνj|HF⟩,1≤i,j≤p. (37)

Now, let’s assume that the left and right eigenvectors are orthonormal,

Lx*Ry*=δxy. (38)

Then, the CC target wave function can be parameterized according to

|CC〉=eT|HF〉=eT*+δT|HF〉=eδT|CC*〉, (39)

where the cluster operator is split into the parent-space component and the correction that arises due to the presence of the auxiliary space,

T=T*+δT, (40)

with the correction term affecting both the parent and the auxiliary space,

$$\delta T=\overset{t}{\underset{i=1}{\sum }}\underset{\mu_{i}}{\sum }\delta t_{\mu_{i}}\theta_{\mu_{i}}.$$ (41)

The target state amplitudes are split in an analogous manner,

tμi=tμi*+δtμi,1≤i≤t, (42)

where the parent space amplitudes have a vanishing auxiliary excitation space component

tμi*=0,p<i≤t. (43)

Thus, the amplitude equations become

Ωμi=⟨μi|e−δTH0T*eδT|HF⟩=0,1≤i≤t. (44)

Finally, the expression for the Jacobian of the target state reads

Jμiνj=⟨μi|e−δTH0T*eδT,θνj|HF⟩,1≤i,j≤t. (45)

The corrections to the excitation energy of the CC parent state are derived by an expansion of the different components that enter the CC target state eigenvalue problem in Eq. 2) in orders of the similarity-transformed fluctuation potential, ΦT* .

To this end, the ground-state parent-state cluster amplitudes are initially expanded in the orders of Φ*^T^,

$$t_{\mu_{i}}=t_{\mu_{i}}^{\left(0\right)}+\delta t_{\mu_{i}}^{\left(1\right)}+\delta t_{\mu_{i}}^{\left(2\right)}+\cdots \;,\;1\leq i\leq t$$ (46)

with

tμi0=tμi*. (47)

An analogous expansion is applied to the Jacobian,

$$J_{\mu_{i}\nu_{j}}=J_{\mu_{i}\nu_{j}}^{\left(0\right)}+J_{\mu_{i}\nu_{j}}^{\left(1\right)}+J_{\mu_{i}\nu_{j}}^{\left(2\right)}+\cdots \;,\;1\leq i,j\leq t,$$ (48)

to the right excited-state vector

$$R^{x}=R^{x\left(0\right)}+R^{x\left(1\right)}+R^{x\left(2\right)}+\cdots$$ (49)

with

Rx0=Rx*, (50)

and the corresponding excitation energy

$$\omega_{x}=\omega_{x}^{\left(0\right)}+\omega_{x}^{\left(1\right)}+\omega_{x}^{\left(2\right)}+\cdots$$ (51)

with

ωx0=ωx*. (52)

One of the key concepts of the CP theory is that the zeroth-order Jacobian, *J* ^(0)^, in Eq. 48 does incorporate a term that is of first order in Φ*^T^. (This is similar to the “pseudo-perturbation” concept introduced, for example, in the derivation of the non-iterative second-order correction to the random-phase approximation excitation energies. (Christiansen et al., 1998) By this particular choice, the zeroth-order Jacobian, *J* ^(0)^, is guaranteed to correspond to the Jacobian of the parent-state model. To see this, consider the CC extended-parent-state Jacobian,

Jμiνj*=⟨μi|H0T*,θνj|HF⟩i,j=1,2,…,t, (53)

Notice that Jμiνj* has the same structure as the Jacobian of the parent space, Jμiνjp in Eq. 37, but the difference is that the indices *i* and *j* run for Jμiνj* over the entire target space rather than just the parent space (hence the word “extended” in the name of Jμiνj* ). The extended-parent-state Jacobian may now be split into the zeroth- and first-order terms,

J*=J0+J1, (54)

where the zeroth-order part does contain a Φ*^T^ term.

$$J^{\left(0\right)}=\left(\begin{array}{cc} \left\langle \mu_{P}|\left[H_{0}^{*T},\theta_{\nu_{P}}\right]|HF\right\rangle & 0\\ 0 & \left\langle \mu_{A}|\left[f^{*T},\theta_{\nu_{A}}\right]|HF\right\rangle \end{array}\right)$$ (55a)

$$J^{\left(1\right)}=\left(\begin{array}{cc} 0 & \left\langle \mu_{P}|\left[\Phi^{*T},\theta_{\nu_{A}}\right]|HF\right\rangle \\ \left\langle \mu_{A}|\left[\Phi^{*T},\theta_{\nu_{P}}\right]|HF\right\rangle & \left\langle \mu_{A}|\left[\Phi^{*T},\theta_{\nu_{A}}\right]|HF\right\rangle \end{array}\right),$$ (55b)

where the P and A subscripts denote the parent- and auxiliary-space components, respectively.

We have illustrated (with the excitation-energy example) the conceptual foundations of the CP theory, and showed how these foundations guarantee that the zeroth order wave function and its properties are those of the parent excitation space. At infinite order, the target state and its properties are formally recovered.

In practice, the target-state quality is usually recovered at third order (Baudin et al., 2019). The excitation energy corrections through third order become:

$$\omega_{x}^{\left(1\right)}=0,$$ (56)

ωx2=∑q=p+1t⟨Lx*|ΦT*,δTq1,Rx*|HF⟩+⟨Lx*|ΦT*,Rx1|HF⟩, (57)

ωx3=⟨Lx*|ΦT*,δTq2,Rx*|HF⟩+12∑q,r=p+1t⟨Lx*|ΦT*,δTq1,δTr1,Rx*|HF⟩+∑q=p+1t⟨Lx*|ΦT*,δTq1,Rx1|HF⟩+⟨Lx*|ΦT*,Rx2|HF⟩. (58)

The quantities needed to determine excitation energies through third order are collected in Table 4.

**TABLE 4:** The cluster amplitudes, the Jacobian and its right eigenvector through third order. $\varepsilon_{\mu_{i}}$ denotes the orbital energy difference between orbitals that differ in the |HF⟩ and |*μ*_*i*_⟩ determinants. *S*_*ip*_ is a step function, which vanishes for *i* ≤ *p* and equals 1 otherwise.


$\delta t_{\mu_{i}}^{\left(1\right)}$	= 0	1 ≤ *i* ≤ *p*
$\varepsilon_{\mu_{i}}\delta t_{\mu_{i}}^{\left(1\right)}$	$=-\left\langle \mu_{i}|\Phi^{*T}|HF\right\rangle$	*p* < *i* ≤ *t*
$\sum_{j=1}^{p}\sum_{\nu_{j}}J_{\mu_{i}\nu_{j}}^{P}\delta t_{\nu_{j}}^{\left(2\right)}$	$=-\sum_{j=p+1}^{t}\sum_{\nu_{j}}\left\langle \mu_{i}|\left[\Phi^{*T},\theta_{\nu_{j}}\right]|HF\right\rangle \delta t_{\nu_{j}}^{\left(1\right)}$	1 ≤ *i* ≤ *p*
$\varepsilon_{\mu_{i}}\delta t_{\mu_{i}}^{\left(2\right)}$	$=-\sum_{j=p+1}^{t}\sum_{\nu_{j}}\left\langle \mu_{i}|\left[\Phi^{*T},\theta_{\nu_{j}}\right]|HF\right\rangle \delta t_{\nu_{j}}^{\left(1\right)}$	*p* < *i* ≤ *t*
$J_{\mu_{i}\nu_{j}}^{\left(0\right)}$	$=\left\langle \mu_{i}|\left[H_{0}^{*T},\theta_{\nu_{j}}\right]|HF\right\rangle \left(1-S_{ip}\right)\left(1-S_{jp}\right)$ $+\varepsilon_{\nu_{j}}\delta_{\mu_{i}\nu_{j}}S_{ip}S_{jp}$	1 ≤ *i*, *j* ≤ *t*
$J_{\mu_{i}\nu_{j}}^{\left(1\right)}$	$=\left\langle \mu_{i}|\left[\Phi^{*T},\theta_{\nu_{j}}\right]|HF\right\rangle \left(1-S_{ip}\right)S_{jp}$ $+\left\langle \mu_{i}|\left[\Phi^{*T},\theta_{\nu_{j}}\right]|HF\right\rangle S_{ip}\left(1-S_{jp}\right)$ $+\left\langle \mu_{i}|\left[\Phi^{*T},\theta_{\nu_{j}}\right]|HF\right\rangle S_{ip}S_{jp}$	1 ≤ *i*, *j* ≤ *t*
$J_{\mu_{i}\nu_{j}}^{\left(2\right)}$	$=\sum_{q=p+1}^{t}\left\langle \mu_{i}|\left[\left[\Phi^{*T},\delta T_{q}^{\left(1\right)}\right],\theta_{\nu_{j}}\right]|HF\right\rangle$	1 ≤ *i*, *j* ≤ *t*
$J_{\mu_{i}\nu_{j}}^{\left(3\right)}$	$=\sum_{q=1}^{t}\left\langle \mu_{i}|\left[\left[\Phi^{*T},\delta T_{q}^{\left(2\right)}\right],\theta_{\nu_{j}}\right]|HF\right\rangle$ $+\frac{1}{2}\sum_{q,r=p+1}^{t}\left\langle \mu_{i}|\left[\left[\left[\Phi^{*T},\delta T_{q}^{\left(1\right)}\right],\delta T_{r}^{\left(1\right)}\right],\theta_{\nu_{j}}\right]|HF\right\rangle$	1 ≤ *i*, *j* ≤ *t*
$R_{\nu_{j}}^{x\left(1\right)}$	= 0	1 ≤ *i* ≤ *p*
(εμi−ωx*)Rνjx(1)	=−⟨μi|[ΦT*,Rx*]|HF⟩	*p* < *i* ≤ *t*
∑j=1p∑νj(JμiνjP−ωx*δμiνj)Rνjx(2)	=ωx(2)Rx*−∑q=p+1t⟨μi|[[ΦT*,δTq(1)],Rx*]|HF⟩ −⟨μi|[ΦT*,Rx(1)]|HF⟩	1 ≤ *i* ≤ *p*
(εμi−ωx*)Rνjx(2)	=−∑q=p+1t⟨μi|[[ΦT*,δTq(1)],Rx*]|HF⟩ +⟨μi|[ΦT*,Rx(1)]|HF⟩	*p* < *i* ≤ *t*

### 6.3 A strategy for massively parallel implementations of cluster perturbation theory in singles and doubles excitation space

As discussed in (Baudin et al., 2019), CP theory excitation energies have been explicitly derived up to the third order for a CC singles parent excitation space and a doubles auxiliary excitation space in the CPS(D-3) model. A significant innovation over traditional methods is that the calculations of $\omega_{x}^{\left(3\right)}$ are non-iterative in the doubles excitation space. The same, of course, pertains to $\omega_{x}^{\left(2\right)}$ , which turns out to be identical to the well-known CIS(D) model (Head-Gordon et al., 1994). Furthermore, for computing excitation energies, the CP framework allows contributions to $\omega_{x}^{\left(2\right)}$ and $\omega_{x}^{\left(3\right)}$ from batches over a virtual molecular orbital index to be determined independently and subsequently summed, which greatly enhances the efficiency and parallelizability of the method. In the following sections, the most crucial aspects of the CP theory implementation for the excitation energies are discussed. For a more extensive and thorough insight into the algorithm, including performance, wall times, computational cost, and various numerical tests, the reader is encouraged to consult (Baudin et al., 2019; Hillers-Bendtsen et al., 2023).

### 6.4 The singlet biorthonormal basis

Efficient parallelization of the third-order excitation energy correction, *ω* ^(3)^, in the CP method requires the use of singlet biorthonormal basis working equations. By expressing the excitation energy corrections to the coupled cluster singles (CCS) excitation energy in singlet biorthonormal basis, the working equations for the second-order correction of the CPS(D-3) excitation energy model (Baudin et al., 2019) can be obtained as

$$\begin{array}{ll} \omega_{x}^{\left(2\right)} & =-\underset{ai}{\sum }L_{ai}^{\mathrm{CCS}}\left[\underset{\mathrm{jckd}}{\sum }R_{di}^{\mathrm{CCS}}t_{\mathrm{ajck}}^{\left(1\right)}L_{\mathrm{kcjd}}+\underset{\mathrm{bjdl}}{\sum }R_{dl}^{\mathrm{CCS}}{\tilde{t}}_{\mathrm{aibj}}^{\left(1\right)}L_{\mathrm{jbld}}\right.\\ & \;\left.-\underset{\mathrm{bjcl}}{\sum }R_{al}^{\mathrm{CCS}}t_{\mathrm{bjci}}^{\left(1\right)}L_{\mathrm{jblc}}+\underset{\mathrm{bck}}{\sum }{\tilde{R}}_{\mathrm{bick}}^{\left(1\right)}\left(kc|ab\right)-\underset{\mathrm{jck}}{\sum }{\tilde{R}}_{\mathrm{ajck}}^{\left(1\right)}\left(kc|ji\right)\right], \end{array}$$ (59)

where *L*_*pqrs*_ = 2 (*pq*|*rs*) − (*ps*|*rq*), and the barred integrals may be represented by

$$\left(pq\bar{|}rs\right)={\hat{P}}_{qs}^{pr}\underset{\alpha \beta \gamma \delta }{\sum }\left({\bar{X}}_{\alpha p}C_{\beta q}+C_{\alpha p}{\bar{Y}}_{\beta q}\right)C_{\gamma r}C_{\delta s}\left(\alpha \beta |\gamma \delta \right),$$ (60)

with.

$${\bar{X}}_{\alpha i}=0,\;\;{\bar{X}}_{\alpha a}=-\underset{i}{\sum }C_{\alpha i}R_{ai}^{\mathrm{CCS}},$$ (61a)

$${\bar{Y}}_{\alpha i}=\underset{a}{\sum }C_{\alpha a}R_{ai}^{\mathrm{CCS}},\;{\bar{Y}}_{\alpha a}=0,$$ (61b)

where the i,j,k,l and a,b,c,d indices denote molecular orbitals (MOs) that are, respectively, occupied and unoccupied in the reference Hartree-Fock state. The Greek indices pertain to the atomic-orbital (AO) basis and *C*_*α*_ *p* are AO-to-MO transformation coefficients. Adopting the conventional approach for computing the tensor elements (Helgaker et al., 2000), the first-order cluster amplitudes read

$${\tilde{t}}_{\mathrm{aibj}}^{\left(1\right)}=\frac{2\left(ai|jb\right)-\left(aj|ib\right)}{\varepsilon_{i}-\varepsilon_{a}+\varepsilon_{j}-\varepsilon_{b}},$$ (62)

whereas the first-order right eigenvector is symmetrized and written in the singlet basis as

$${\tilde{R}}_{\mathrm{aibj}}^{\left(1\right)}=\frac{2\left(ai\bar{|}jb\right)-\left(aj\bar{|}ib\right)}{\varepsilon_{i}-\varepsilon_{a}+\varepsilon_{j}-\varepsilon_{b}+\omega^{\mathrm{CCS}}}.$$ (63)

The third-order excitation energy correction may be formulated via

$$\omega_{x}^{\left(3\right)}=\underset{\mathrm{aibj}}{\sum }{\tilde{t}}_{\mathrm{aibj}}^{\left(2\right)}\left[{\hat{P}}_{ij}^{ab}R_{ai}^{\mathrm{CCS}}{\bar{F}}_{jb}-\left(ia\overset{˘}{|}jb\right)\right]+\underset{\mathrm{aibj}}{\sum }\left[{\tilde{R}}_{\mathrm{aibj}}^{\left(2\right)}+2R_{ai}^{\mathrm{CCS}}t_{bj}^{\left(2\right)}-R_{aj}^{\mathrm{CCS}}t_{bi}^{\left(2\right)}\right]\left(ai\bar{|}bj\right),$$ (64)

where the expression involving the two-electron integral is defined by

$$\left(ia\overset{˘}{|}jb\right)={\hat{P}}_{ab}^{ij}\underset{\alpha \beta \gamma \delta }{\sum }\left({\overset{˘}{X}}_{\alpha i}C_{\beta a}+C_{\alpha i}{\overset{˘}{Y}}_{\beta a}\right)C_{\gamma j}C_{\delta b}\left(\alpha \beta |\gamma \delta \right),$$ (65)

with

$${\overset{˘}{X}}_{\alpha i}=\underset{j}{\sum }C_{\alpha j}\underset{b}{\sum }L_{bi}^{\mathrm{CCS}}R_{bj}^{\mathrm{CCS}}=\underset{j}{\sum }C_{\alpha j}D_{ij}^{\mathrm{CCS}}$$ (66)

$${\overset{˘}{Y}}_{\alpha a}=\underset{b}{\sum }C_{\alpha b}\underset{j}{\sum }L_{aj}^{\mathrm{CCS}}R_{bj}^{\mathrm{CCS}}=\underset{j}{\sum }C_{\alpha b}D_{ab}^{\mathrm{CCS}}.$$ (67)

Finally, the second-order doubles cluster amplitudes and right eigenvectors may be expressed as

$${\tilde{t}}_{\mathrm{aibj}}^{\left(2\right)}=\frac{2X_{\mathrm{aibj}}^{t}-X_{\mathrm{ajbi}}^{t}}{\varepsilon_{i}-\varepsilon_{a}+\varepsilon_{j}-\varepsilon_{b}}$$ (68)

and

$${\tilde{R}}_{\mathrm{aibj}}^{\left(2\right)}=\frac{2X_{\mathrm{aibj}}^{R}-X_{\mathrm{ajbi}}^{R}}{\varepsilon_{i}-\varepsilon_{a}+\varepsilon_{j}-\varepsilon_{b}},$$ (69)

where the second order intermediates $X_{\mathrm{aibj}}^{t}$ and $X_{\mathrm{aibj}}^{R}$ are given in Tables 5 and VI of Ref. (Baudin et al., 2019).

**TABLE 5:** The first 5 roots for the retinal excited states, all values in eV.

Root	CCS	CPS(D-2)	CPS(D-3)
1	3.274	2.370	2.498
2	5.038	3.621	3.930
3	6.105	4.650	4.874
4	6.161	4.443	4.560
5	6.993	5.388	5.590

### 6.5 The multi-node multi-GPU accelerated CPS(D-3)

The implementation of the CPS(D-3) is based on the RI approximation (Vahtras et al., 1993; Hättig and Weigend, 2000) for two-electron repulsion integrals, which facilitates the efficient calculation of *ω* ^(3)^ in batches over a virtual index. The RI approximation is achieved by constructing three-center integral fitting coefficients from an auxiliary basis set using the Coulomb metric:

$$B_{pq}^{P}=\underset{P}{\sum }\left(pq|P\right){\left(P|Q\right)}^{-1/2}.$$ (70)

This allows the four-index two-electron repulsion integrals to be represented by

$$\left(pq|rs\right)=\underset{PQ}{\sum }\left(pq|P\right){\left(P|Q\right)}^{-1}\left(Q|rs\right)=\underset{P}{\sum }B_{pq}^{P}B_{rs}^{P}.$$ (71)

The three-index fitting coefficients obtained from the RI approximation can be written to disk and read into memory in batches over a virtual index. This enables the calculation of the third-order excitation energy correction using a massively parallel algorithm that can efficiently adapt to the available computational resources.

By calculating the incremental contributions to *ω* ^(3)^ independently and on-demand for each batch, the need to store fourth-order tensors in main memory and recalculate the first-order doubles amplitudes $t_{2}^{\left(1\right)}$ is eliminated. This allows for scalability of the batch size according to the available memory. The main CPS(D-3) algorithm is summarized by Algorithm 1.

Algorithm 1 Massively Parallel Algorithm for Calculating the Third Order Excitation Energy Correction to CCS via the CPS(D-3) model.

 **Step 1:** Initialize transformation matrices, integral fitting coefficients, and Fock matrices
 **Step 2:** Calculate fully virtual three index integral fitting coefficients and distribute in global memory
**for** *A (batch over virtual index a)* **do**
 **Step 3.1:** Calculate second order doubles intermediate $X_{\mathrm{Aibj}}^{t}$

 **Step 3.2:** Calculate $\left(iA\overset{˘}{|}jb\right)$

 **Step 3.3:** Update excitation energy contribution, $\omega^{\left(3\right)}=\omega^{\left(3\right)}+\sum_{a\in A}\sum_{\mathrm{ibj}}\frac{\left(2X_{\mathrm{aibj}}^{t}-X_{\mathrm{ajbi}}^{t}\right)}{\varepsilon_{i}-\varepsilon_{a}+\varepsilon_{j}-\varepsilon_{b}}\left({\hat{P}}_{ij}^{ab}R_{ai}^{\mathrm{CCS}}{\bar{F}}_{jb}-\left(ia\overset{˘}{|}jb\right)\right)$

**end for**
 **Step 4:** Deallocate $B_{\overset{˘}{i}a}^{P}$ and $B_{i\overset{˘}{a}}^{P}$ from memory
 **Step 5:** Calculate three center integral fitting coefficients $B_{\bar{a}i}$ , $B_{a\bar{i}}$ , and $B_{\bar{i}j}$

**for** *A (batch over virtual index a)* **do**
 **Step 6.1:** Calculate second order doubles intermediate $X_{\mathrm{Aibj}}^{R}$

 **Step 6.2:** Calculate $\left(Ai\bar{|}bj\right)$

 **Step 6.3:** Update excitation energy contribution, $\omega^{\left(3\right)}=\omega^{\left(3\right)}+\sum_{a\in A}\sum_{\mathrm{ibj}}\left(\frac{\left(2X_{\mathrm{aibj}}^{R}-X_{\mathrm{ajbi}}^{R}\right)}{\varepsilon_{i}-\varepsilon_{a}+\varepsilon_{j}-\varepsilon_{b}}+2R_{ai}^{\mathrm{CCS}}t_{bj}^{\left(2\right)}-R_{aj}^{\mathrm{CCS}}t_{bi}^{\left(2\right)}\right)\left(Ai\bar{|}bj\right)$

**end for**

The CPS(D-3) algorithm described in Algorithm 1 is parallelized for distributed memory architectures using a master-worker model and MPI. The algorithm for the *ω* ^(3)^ calculation is initiated by the master rank, which determines the transformation matrices $\bar{X}$ , $\bar{Y}$ , $\overset{˘}{X}$ , and $\overset{˘}{Y}$ . Next, the algorithm calculates the three-center integral fitting coefficients, $B_{ai}^{P}$ , $B_{ij}^{P}$ , $B_{\overset{˘}{i}a}^{P}$ , and $B_{i\overset{˘}{a}}^{P}$ , followed by the one-index transformed Fock matrices, $\bar{F}ia$ , $\bar{F}{ij}^{\prime }$ , and $\bar{F}{ab}^{\prime }$ . Finally, the fully virtual three-index integral fitting coefficients, *Bab* ^*P*^ and $B_{\bar{a}b}^{P}$ , are determined. Once these quantities are determined by the master rank, they are broadcasted to all other ranks. To distribute the fully virtual three index integral fitting coefficients, tiled distributed tensors are utilized through the Scalable Tensor Library (ScaTeLib) (Ettenhuber, 2023). Subsequently, the master rank assigns the calculation of the first contributions to *ω* ^(3)^ to the other ranks, to be computed in batches over the virtual index *a*. These ranks execute the computation of the contribution to *ω* ^(3)^ for a particular batch and return the incremental contribution. The master rank then accumulates these incremental contributions to determine the total *ω* ^(3)^ correction. After all workers complete the first loop of Algorithm 1, the master node identifies a new set of three center integral fitting coefficients required for the second contribution to *ω* ^(3)^, which is computed in the second loop of Algorithm 1. The master rank then assigns the computation of the second contribution to *ω* ^(3)^ to the other ranks in batches over the virtual index *a*. This process leads to the determination of the full *ω* ^(3)^ correction.

In the primary algorithm of the CPS(D-3) implementation, Algorithm 2 is employed to calculate the $X_{\mathrm{aibj}}^{t}$ intermediates for a given batch *A*. Minor modifications are made to utilize the algorithm for the computation of $X_{\mathrm{aibj}}^{R}$ ; Algorithm 2 begins by calculating the first-order correction for the doubles excitation amplitudes, $t_{\mathrm{Aidk}}^{\left(1\right)}$ . It then retrieves the tiled distributed tensor $B_{cA}^{P}$ . The algorithm proceeds with two nested loops, iterating over virtual index *D* in batches, and occupied index *L* in batches. In the loop over virtual index *D*, several intermediate values are computed, such as $t_{\mathrm{AiDk}}^{\left(1\right)}$ (*bD*|*kj*) (*cA*|*bD*), $t_{\mathrm{AkDj}}^{\left(1\right)}$ , and $t_{\mathrm{Djci}}^{\left(1\right)}$ . These values are used to iteratively update the $X_{\mathrm{Aibj}}^{t}$ . Once this loop is completed, the algorithm proceeds with the loop over the occupied index *L*. Here, $t_{\mathrm{AkbL}}^{\left(1\right)}$ and (*ik*|*jL*) are calculated and used to further update the $X_{\mathrm{Aibj}}^{t}$ value. After completing the loop over *L*, the algorithm calculates $Y_{Ai}^{P}$ outside of the loops and uses it to finalize the update of the $X_{\mathrm{Aibj}}^{t}$ value. This method allows for an efficient and systematic computation of the first-order correction to the doubles excitation amplitudes and the $X_{\mathrm{aibj}}^{t}$ (or $X_{\mathrm{aibj}}^{R}$ ) value for a given batch *A*, accounting for the contributions from both occupied and virtual indices in the process.

Algorithm 2 Algorithm for calculating the intermediate $X_{\mathrm{aibj}}^{t}$ for a given batch *A*.

 **Step 1:** Calculate first-order correction for doubles excitation amplitudes, $t_{\mathrm{Aidk}}^{\left(1\right)}=\left(Ai|dk\right)/\left(\varepsilon_{i}-\varepsilon_{A}+\varepsilon_{k}-\varepsilon_{d}\right)$

 **Step 2:** Retrieve tiled distributed tensor $B_{cA}^{P}$

**for** *D (batch over virtual index)* **do**
 **Step 3.1:** Extract $t_{\mathrm{AiDk}}^{\left(1\right)}$ from $t_{\mathrm{Aidk}}^{\left(1\right)}$

 **Step 3.2:** Get tiled distributed tensor $B_{bD}^{P}$

 **Step 3.3:** Calculate (*bD*|*kj*)
 **Step 3.4:** Update $X_{\mathrm{Aibj}}^{t}$ using $t_{\mathrm{AiDk}}^{\left(1\right)}$ and (*bD*|*kj*)
 **Step 3.5:** Update $X_{\mathrm{Aibj}}^{t}$ using $t_{\mathrm{AkDj}}^{\left(1\right)}$ and (*bD*|*ki*)
 **Step 3.6:** Calculate (*cA*|*bD*)
 **Step 3.7:** Calculate first-order correction for $t_{\mathrm{Djci}}^{\left(1\right)}$

 **Step 3.8:** Update $X_{\mathrm{Aibj}}^{t}$ using $t_{\mathrm{Djci}}^{\left(1\right)}$ and (*cA*|*bD*)
**end for**
**for *L*** *(batch over occupied index)* **do**
 **Step 4.1:** Extract $t_{\mathrm{AkbL}}^{\left(1\right)}$ from $t_{\mathrm{Akbl}}^{\left(1\right)}$

 **Step 4.2:** Calculate (*ik*|*jL*)
 **Step 4.3:** Update $X_{\mathrm{Aibj}}^{t}$ using $t_{\mathrm{AkbL}}^{\left(1\right)}$ and (*ik*|*jL*)
**end for**
 **Step 5:** Calculate $Y_{Ai}^{P}$ using the given summation formula
 **Step 6:** Update the $X_{\mathrm{Aibj}}^{t}$ value using the calculated values of $Y_{Ai}^{P}$ and $B_{bj}^{P}$

Algorithm 1; Algorithm 2 provide a scheme that enables the calculation of CPS(D-3) excitation energies for system sizes beyond the reach of conventional CCSD calculations. To perform all the costly tensor contractions in each batch, these algorithms use the Tensor Algebra Library for Shared Memory Computers (TALSH) (Lyakh, 2023), which transfers them to GPUs. Open Multiprocessing (OMP) allows each rank to exploit shared memory parallelism locally. Moreover, the computation of $\left\langle \mu_{1}|\left[\Phi ,T_{2}^{\left(1\right)}\right]|HF\right\rangle$ is parallelized as the $O\left(N^{5}\right)$ scaling of this term is a bottleneck for larger systems.

### 6.6 Numerical illustrations: CPS(D-3) excitation energies

**FIGURE 4:**
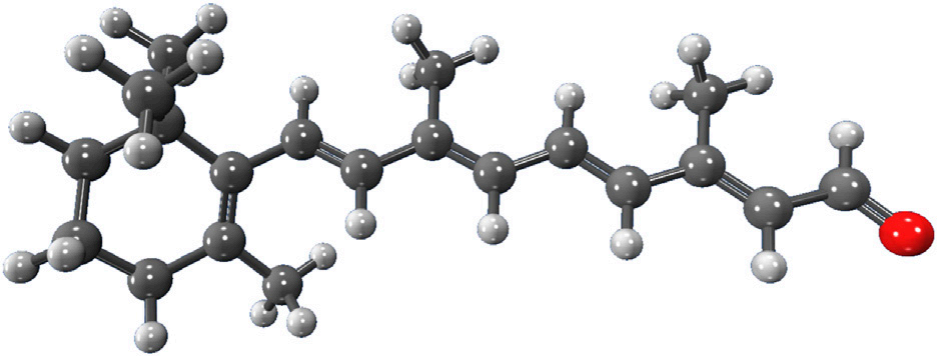
Retinal molecule: 63 atoms, 540 basis functions and 1932 auxiliary basis functions.

In the case of CPS(D-n) methods, the retinal molecule (see Figure 4) with a cc-pVDZ basis set serves as an excellent example of a molecule with degenerate orbitals and delocalized electrons. This molecule is also of significant importance due to its biological role in photochemical reactions. The retinal system contains 63 atoms, and for the demonstrative calculation, 540 basis functions and 1932 auxiliary basis functions were employed. The calculation was performed using the GPU-accelerated LS-Dalton implementation on the Summit supercomputer at Oak Ridge National Laboratory. The first five roots, corresponding to the first excitation energies, are summarized in the table below.

The total time for the CPS(D-3) calculation on the retinal molecules was less than 20 min using 64 MPI processes. This balance between wall time and computational resources makes approximations such as CPS(D-3) practical tools capable of providing experimental molecular scientists with chemical observables at an accuracy of the CC target model (in this case, CCSD).

## 7 Discussion and outlook

The DEC scheme presents a divide-and-conquer linear-scaling and massively parallel framework for CC calculations, assuring error control in a black-box manner. These characteristics make DEC an enticing computational basis for molecular modeling on extensive molecular systems. Specifically, the DEC scheme imparts CC methods with computational abilities that are generally unattainable with conventional CC algorithms. Nevertheless, the DEC framework also faces challenges, such as the high DEC prefactor resulting from the need to recalculate integrals and amplitudes due to overlapping orbital spaces across different fragments.

In single compute node calculations, the crossover point in computational effort between DEC and a traditional, canonical calculation arises in large molecular systems. If a canonical calculation is achievable, it is likely more efficient than the DEC calculation. However, when multiple compute nodes are available, the massively parallel nature of the DEC algorithm leads to a reduced time-to-solution compared to a canonical calculation. As floating point operations continue to become more cost-effective and the number of cores on a compute node increases, parallelization will be crucial, and a considerable amount of recalculation will be acceptable if it facilitates a massively parallel computational strategy.

The DEC scheme, in principle, lays the foundation for linear-scaling and massively parallel implementations of any CC model. However, multiple technical challenges must be addressed before DEC can become a mainstream tool for more accurate CC models. For example, although the error control of the DEC-CCSD(T) method has been tackled, the current DEC-CCSD(T) algorithm can only be applied to large molecules for loose FOT values due to the fragment sizes encountered. When analyzing large molecules with a triple-*ζ* quality basis set, the resulting fragments often contain over 1000 basis functions, making such calculations impractical even for massively parallel CCSD(T) implementations and hindering high-accuracy DEC-CCSD(T) applications. The large pair fragments, however, are frequently associated with minor energy contributions, and it may be possible to further reduce the pair orbital spaces without sacrificing the accuracy of the final correlation energy. Alternatively, the scaling of high-level CC fragment calculations could be decreased by considering tensor factorization techniques, PNOs, or other fragment-specific orbitals. Combining the DEC scheme with PNO-based local CC methods, for instance, would fully harness the sparsity of correlation effects in each fragment calculation, enabling CC calculations on systems of unprecedented sizes by removing all bottlenecks of wave function-based approximations through the DEC scheme—provided that the HF solver which generates the underlying reference is implemented with comparable efficiency.

Lastly, it is essential to note that the implementation of the DEC scheme ensures performance portability, allowing the DEC scheme to automatically benefit from new hardware developments and expand its application range as computational resources become more accessible. Although this work anticipates a promising future for DEC CC calculations, it is crucial to remember that many chemistry applications require energy differences rather than total energies. In these scenarios, computational chemistry practitioners should concentrate on direct approaches, such as CP theory.

CP theories provide a promising potential in addressing the limitations of total energy calculations with DEC for large molecular systems. The systematic approach offered by CP theories allows for the accurate calculation of excitation energies and other molecular properties at a CCSD level, providing a more robust, efficient and direct way to understand and predict the behavior of complex molecular systems.

The development of the CPS(D-3) model, as discussed in the literature, serves as a significant advancement in this area. By employing perturbation corrections up to the third order within the CP framework, the CPS(D-3) model delivers excitation energies of CCSD quality. The non-iterative nature of the $\omega_{x}^{\left(2\right)}$ and $\omega_{x}^{\left(3\right)}$ correction calculations in the doubles excitation space enables a massively parallel implementation, distributing batches over a virtual molecular orbital index across different compute nodes using the MPI.

The application of modern heterogeneous supercomputers and GPUs for accelerating heavy tensor contractions further enhances the efficiency and scalability of the CPS(D-3) calculations. This approach makes it feasible to calculate high quality excitation energies for molecular systems with several thousand basis functions, given sufficient computational resources.

Moreover, CP theory demonstrates versatility in its potential applications, extending to the calculation of other time-independent and time-dependent properties (Pawłowski et al., 2019c; Hillers-Bendtsen et al., 2022). This adaptability paves the way for the development of similar massively parallel implementations in other areas of research.

## Data Availability

The raw data supporting the conclusion of this article will be made available by the authors, without undue reservation.

## Footnotes

1 The asterisk in the equation should not be confused with complex conjugation.
