# Corrigendum: Coupled cluster theory on modern heterogeneous supercomputers

**DOI:** 10.3389/fchem.2023.1256510

**Published:** 2023-08-15

**Authors:** Hector H. Corzo, Andreas Erbs Hillers-Bendtsen, Ashleigh Barnes, Abdulrahman Y. Zamani, Filip Pawłowski, Jeppe Olsen, Poul Jørgensen, Kurt V. Mikkelsen, Dmytro Bykov

**Affiliations:** ^1^ Oak Ridge National Laboratory, Oak Ridge, TN, United States; ^2^ Department of Chemistry, University of Copenhagen, Copenhagen, Denmark; ^3^ Department of Chemistry and Biochemistry and Center for Chemical Computation and Theory, University of California, Merced, CA, United States; ^4^ Department of Chemistry and Biochemistry, Auburn University, Auburn, AL, United States; ^5^ Department of Chemistry, Aarhus University, Aarhus, Denmark

**Keywords:** coupled cluster theory, divide-expand-consolidate coupled cluster framework, cluster perturbation theory, excitation energies, tetrahydrocannabinol, deoxyribonucleic acid

In the published article, the bibliography file was corrupted. As a consequence there were several inconsistencies within the citation list: titles did not match author lists or journal name or were parsed wrong and added in parts to a citation. Additionally, there were a few references included in the published version of the manuscript that were not a part of the original bibliography. The correct **References** list appears below.

The authors apologize for this error and state that this does not change the scientific conclusions of the article in any way. The original article has been updated.
